# Regulatory dynamics of gene expression in the developing male gametophyte of *Arabidopsis*

**DOI:** 10.1007/s00497-022-00452-5

**Published:** 2022-10-25

**Authors:** Božena Klodová, David Potěšil, Lenka Steinbachová, Christos Michailidis, Ann-Cathrin Lindner, Dieter Hackenberg, Jörg D. Becker, Zbyněk Zdráhal, David Twell, David Honys

**Affiliations:** 1 Institute of Experimental Botany of the Czech Academy of Sciences, Rozvojová 263, 165 02 Prague 6, Czech Republic; 2 Department of Experimental Plant Biology, Faculty of Science, Charles University, Viničná 5, Praha 2, 128 00 Czech Republic; 3 Mendel Centre for Plant Genomics and Proteomics, Central European Institute of Technology, Masaryk University, Kamenice 5, 625 00 Brno, Czech Republic; 4 Instituto de Tecnologia Química e Biológica António Xavier, Universidade Nova de Lisboa (ITQB NOVA), Av. da República, 2780-157 Oeiras, Portugal; 5 Department of Genetics and Genome Biology, University of Leicester, Leicester, LE1 7RH UK; 6 Instituto Gulbenkian de Ciência, Rua da Quinta Grande 6, 2780-156 Oeiras, Portugal; 7 National Centre for Biomolecular Research, Faculty of Science, Masaryk University, Kamenice 5, 625 00 Brno, Czech Republic; 8 KWS SAAT SE & Co. KGaA, Grimsehlstraße 31, 37574 Einbeck, Germany

**Keywords:** *Arabidopsis*, Male gametophyte, RNA-seq, Proteome, Microgametogenesis

## Abstract

Sexual reproduction in angiosperms requires the production and delivery of two male gametes by a three-celled haploid male gametophyte. This demands synchronized gene expression in a short developmental window to ensure double fertilization and seed set. While transcriptomic changes in developing pollen are known for *Arabidopsis*, no studies have integrated RNA and proteomic data in this model. Further, the role of alternative splicing has not been fully addressed, yet post-transcriptional and post-translational regulation may have a key role in gene expression dynamics during microgametogenesis. We have refined and substantially updated global transcriptomic and proteomic changes in developing pollen for two *Arabidopsis* accessions. Despite the superiority of RNA-seq over microarray-based platforms, we demonstrate high reproducibility and comparability. We identify thousands of long non-coding RNAs as potential regulators of pollen development, hundreds of changes in alternative splicing and provide insight into mRNA translation rate and storage in developing pollen. Our analysis delivers an integrated perspective of gene expression dynamics in developing *Arabidopsis* pollen and a foundation for studying the role of alternative splicing in this model.

**Supplementary Information:**

The online version contains supplementary material available at 10.1007/s00497-022-00452-5.

## Introduction

The life cycle of land plants alternates between haploid gametophyte and diploid sporophyte generations. In seed-bearing plants, male and female gametophytes are reduced to only a few cells supported by the maternal sporophyte and the male gametophytes are dispersed as pollen grains. The extreme reduction of the angiosperm male gametophyte to only three cells requires the regulation of gene expression in a short developmental window to enable double fertilization and seed set. Understanding the dynamics of gene expression in pollen is therefore central for understanding reproductive development, its evolution and role in crop productivity (reviewed in Xu et al. 2011; Raggi et al. 2020).

**Fig. 1:**
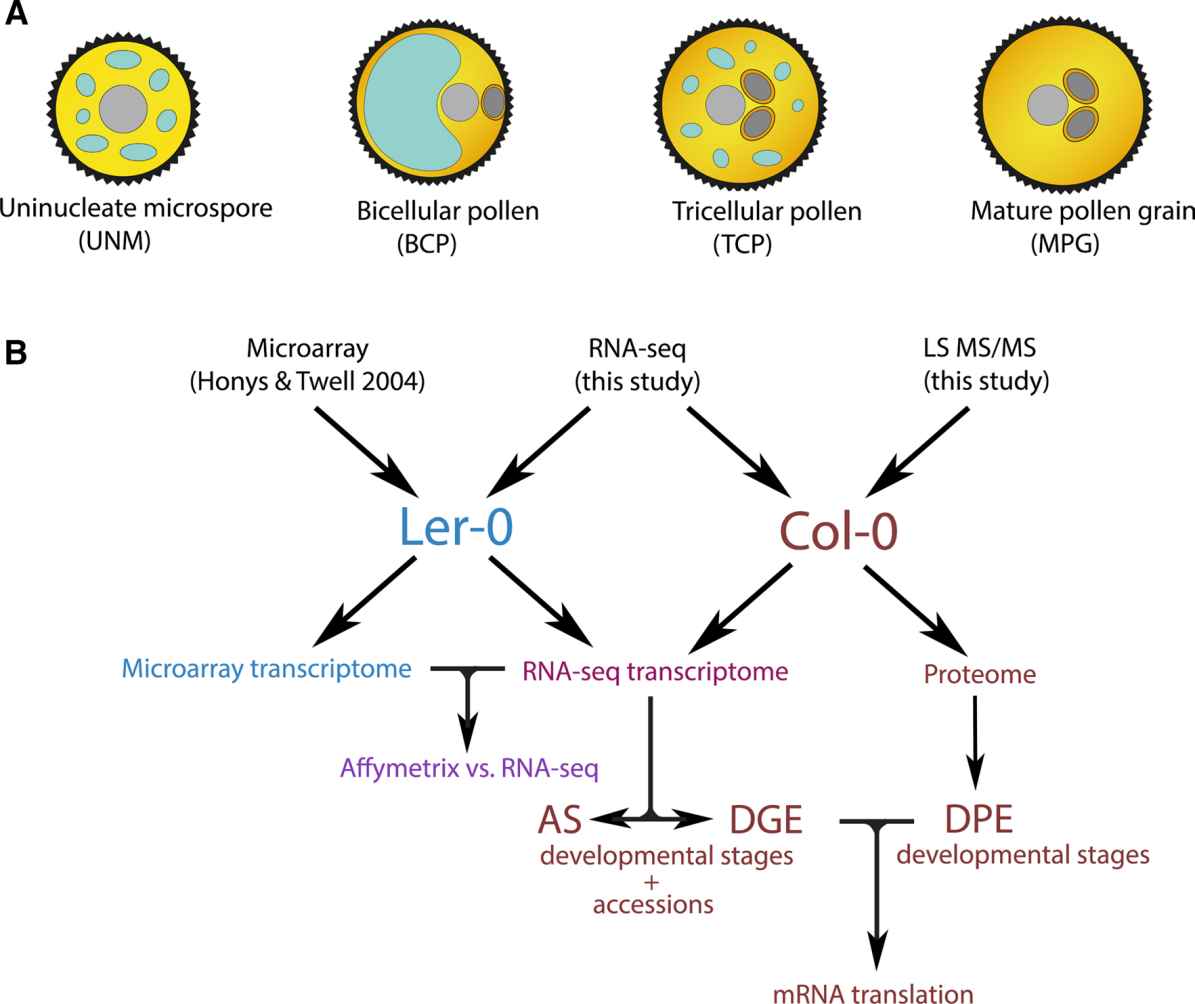
Overview of datasets and analyses. **A** Key stages in *Arabidopsis* microgametogenesis. **B** Datasets used, and analyses executed in this project. AS = alternative splicing, DGE = differential gene expression, DPE = differential protein expression

Male gametophyte development is comprised of two main phases; microsporogenesis, in which diploid microsporocytes undergo meiosis to form tetrads of haploid microspores and microgametogenesis, wherein microspores develop into pollen grains (Fig. 1A). This study is focussed on the second phase, during which uninucleate microspores (UNM) expand and become polarized with the nucleus positioned near the cell wall. Polarized microspores divide asymmetrically at pollen mitosis I (PMI) to form bicellular pollen (BCP), which is comprised of a large vegetative cell and small generative cell. In approximately 30% of angiosperms, including *Arabidopsis thaliana,* the generative cell divides again at pollen mitosis II (PMII) to form tricellular pollen (TCP). Prior to release as a mature pollen grain (MPG), the male gametophyte is partially dehydrated (reviewed in Hackenberg and Twell 2019; Hafidh and Honys 2021). Metabolic re-activation of pollen on the female stigma results in the outgrowth of pollen tubes (PT), which are guided to ovules to deliver twin sperm cells (reviewed in Johnson et al. 2019).

The *Arabidopsis thaliana* mature pollen transcriptome has been analysed using microarray and serial analysis of gene expression (SAGE) methods (Becker et al. 2003; Honys and Twell 2003, 2004; Lee and Lee 2003; Pina et al. 2005). Microarray studies estimated *Arabidopsis* pollen transcriptome complexity to be about 6,000 genes with around 10% pollen-specific genes (Twell et al. 2006). Cell wall, cytoskeleton, signalling and vesicle transport gene ontology (GO) categories are over-represented compared with vegetative tissues, whereas transcription, translation and some metabolic pathways are under-represented. In a landmark study using Affymetrix ATH1 genome arrays, 13,977 *Arabidopsis* male gametophyte-expressed mRNAs were identified, of which 9.7% were considered male gametophyte-specific (Honys and Twell 2004). The developmental transcriptome of pollen undergoes a phase shift in transcript abundance involving a decrease in abundant early transcripts and a corresponding increase of late transcripts. These trends are associated with a reduction of cellular activity and the preferential expression of specific transcript groups during the late developmental phase (Honys and Twell 2003). The results also support the broad division of developmental gene expression into an early, more sporophyte-like phase and a late, more gametophyte-specific phase (Mascarenhas 1990). RNA sequencing (RNA-seq) has provided further insight into the pollen transcriptome of *Arabidopsis* with around 500 newly detected pollen-expressed genes and 2000 previously unannotated splicing events (Loraine et al. 2013). In a recent study, RNA-seq datasets from various organs and gametes of ten plant species were analysed to establish missing components of organogenesis and gamete development in an evolutionary context. There was conservation of the male transcriptome among angiosperms and enrichment of genes with unknown function suggesting undiscovered functions in reproductive development (Julca et al. 2021). Recently, single-cell RNA-seq analysis of microgametogenesis in maize highlighted phase shifts associated with meiotic prophase and the transition from uninucleate microspores to bicellular pollen (Nelms and Walbot 2022).

Studies of the pollen proteome with methods such as gel free liquid chromatography tandem mass spectrometry (LC MS/MS) platforms, have been used to identify changes in protein levels in distinctive conditions, or developmental stages. Studies focussed on mature pollen or pollen tubes of various plants including *Arabidopsis*, lily, tomato, rice and olive have revealed enrichment of proteins connected to metabolism, energy generation and cell structure (reviewed in Fíla et al. 2017). Developmental studies of pollen for two solanaceous crops identified 1821 proteins in tomato (Chaturvedi et al. 2013) and 3888 proteins in tobacco (Ischebeck et al. 2014). Both studies reported dynamic changes in metabolic pathways and identified groups of proteins specific for each developmental stage. In another pollen proteomic study of tomato, groups of mRNAs were identified that differ in the timing of translation under heat stress (Keller et al. 2018).

We have analysed the transcriptome and proteome of the *Arabidopsis* male gametophyte at four developmental stages (Fig. 1A). RNA-seq was used to compare transcriptomic data for Columbia-0 (Col-0) and Landsberg erecta (Ler-0) accessions, and LC–MS/MS proteome data was generated for Col-0 (Fig. 1B). The enhanced resolution and sensitivity of RNA-seq is highlighted by comparative analysis with Affymetrix ATH1 microarray data for identical developmental stages (Honys and Twell 2004; Fig. 1B). Mapping of pollen RNA-seq transcriptomes to the *Arabidopsis* transcriptome (TAIR10) identified hundreds of new mRNA alternative splicing events. Our analyses provide a map of pollen transcriptome dynamics and a catalog of alternative splicing events for two *Arabidopsis* accessions. Proteome analysis provides further insight into post-transcriptional fate during *Arabidopsis* microgametogenesis. Collectively, our study integrates global patterns of gene expression in developing pollen and provides a perspective of transcriptomic variability between two *Arabidopsis* accessions.

## Results

### RNA-seq analysis improves resolution in pollen developmental transcriptomics

We examined transcript profiles throughout male gametophyte development for the *Arabidopsis* Col-0 and Ler-0 accessions using RNA-seq. RNA-seq data were obtained from pure populations of isolated microspores and pollen at four developmental stages: unicellular microspores (UNM), bicellular pollen (BCP), tricellular pollen (TCP), and mature pollen grains (MPG). Three biological replicates were used for each stage. RNA-seq reads were mapped to 33,988 TAIR10 annotated gene models (Berardini et al. 2015) and gene expression was calculated with TPM normalization (Supplementary File 1). In previous analysis using Affymetrix ATH1 Genome Arrays, expression profiles were determined for the same four pollen developmental stages of Ler-0 (Honys and Twell 2004). The ATH1 array harboured probes sets for 22,591 gene models based on the *Arabidopsis* Genome Initiative annotation (GEO accession number: GPL198) and the majority of these (93%, 21,038 gene models) corresponded to genes that mapped in the new RNA-seq datasets.

**Fig. 2:**
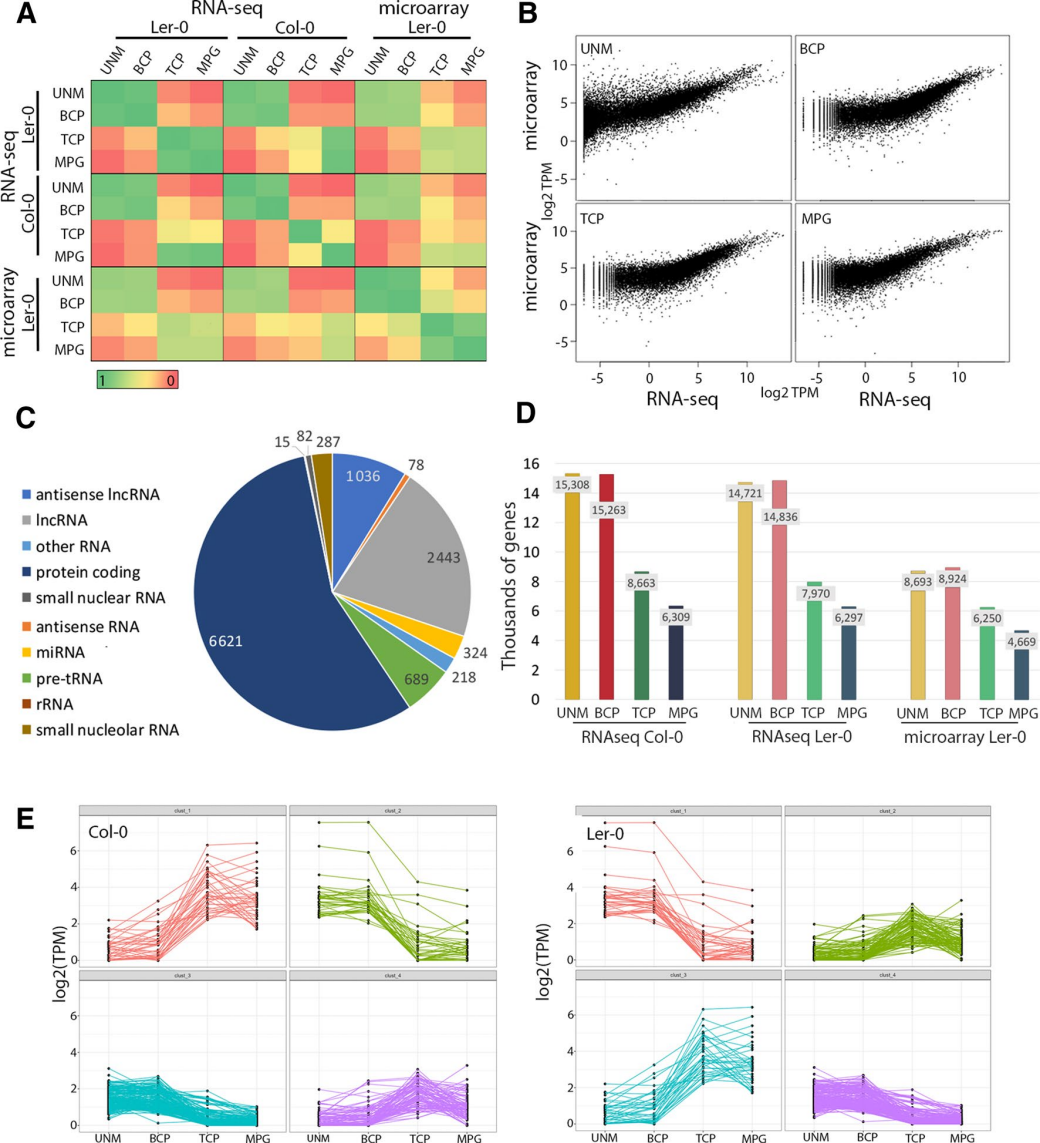
Comparison of RNA-seq and pollen microarray expression data. **A** Pearson correlation (*r*) shows high similarity between profiling methods. Colour bar reflects *r* values. **B** Scatter plot of TPM values (RNA-seq) and signal values (microarray) for the 21,038 gene models present in both analyses showing higher dynamic range for RNA-seq data. **C** Distribution of 12,939 transcripts detected only by RNA-seq. **D** RNA-seq and microarray data show similar trends in the numbers of expressed genes. RNA-seq data shows numbers of genes with expression above 3 TPM. The microarray data shows genes with reliable detection call (8). **E** Expression profiles of lncRNAs in Col-0 (left) and Ler-0 (right) represented by k-means clustering. Major profiles are early expression of lncRNAs in UNM and BCP and late expression in TCP and MPG. Each line represents a single lncRNA and all genes with summed expression for three biological replicates above 3 TPM are plotted

The level of similarity between RNA-seq and microarray data was evaluated by comparison of Pearson correlation coefficients. We observed a positive correlation between corresponding datasets regardless of the platform used (Fig. 2A). RNA-seq and microarray data for Ler-0 were the most similar (*r* > 0.74) for UNM and BCP stages, with lower values (*r* > 0.59) for TCP and MPG. The same trend was seen for Col-0 RNA-seq and Ler-0 microarray data, but with lower correlation coefficients at all stages (Fig. 2A). For both accessions, RNA-seq data showed greater similarity between early developmental stages than to either of the late developmental stages and vice versa, in accord with previous analysis (Honys and Twell 2004; Fig. 2A). Scatter plot comparisons illustrate the similarity between RNA-seq and microarray datasets (Fig. 2B). On the other hand, the S-shaped skewing of the nonlinear regression for genes with extremely high or low expression (Fig. 2B), highlights the higher dynamic range expected for RNA-seq data (Marioni et al. 2008).

We compared the number of genes with detectable expression at each developmental stage with both methods and for both accessions (Fig. 2D). Previous microarray analysis identified 21,038 expressed genes in Ler-0 according to their MAS5.0 present call threshold, representing 8693 genes in UNM, 8924 in BCP, 6250 in TCP and 4669 genes in MPG (Twell et al. 2006). As expected, RNA-seq detected greater numbers of genes for both accessions. In Ler-0, 14,721 genes were detected in UNM, 14,836 in BCP, 7970 in TCP and 6297 in MPG. The numbers of genes in Col-0 were slightly higher, at 15,308 in UNM, 15,263 in BCP, 8663 in TCP and 6309 in MPG. For both datasets, there was substantial reduction in the number of expressed genes at late developmental stages.

RNA-seq data provided evidence for 26,916 expressed genes in one or more stages of developing Ler-0 pollen, which almost doubled the number (13,977) detected by microarray analysis (out of the total 22,591 probes used; Honys and Twell 2004). Of the 12,939 newly detected genes, approximately half (6621, 51.1%) are protein coding RNAs, while a substantial fraction (3479, 26.9%) are long non-coding RNAs (lncRNAs). The remaining 2839 expressed genes (22%) were other non-coding RNAs, such as pre-tRNAs or small nucleolar RNAs (Fig. 2C).

The expression profiles of newly detected pollen-expressed genes were compared to that of all expressed genes. The average expression of newly detected genes was 17.6 TPM in Col-0 and 16.3 TPM in Ler-0, whereas average expression for all gene models (33,988 genes) was 29.4 TPM for both accessions. For newly detected protein-coding genes, expression was higher than for non-coding genes and their average expression was similar to that for all gene models at 27.5 and 25.6 TPM in Col-0 and Ler-0, respectively. GO analysis of the newly detected protein-coding genes revealed only three enriched biological process terms, ‘regulation of protein localization to cell surface’ (40 genes), ‘regulation of double fertilization forming a zygote and endosperm’ (41 genes) and ‘unclassified’ (1758 genes).

We further analysed lncRNAs as a novel transcript category that could not be studied in previous work (Honys and Twell 2004). Out of 3479 lncRNAs, 2443 were annotated as long non-coding RNAs and 1036 as antisense lncRNAs or natural antisense transcripts (NATs), transcribed from the opposite strands of either protein-coding or non-coding genes. The average expression signals of all lncRNAs were low in both accessions (2.5 TPM in Col-0; 2.6 TPM in Ler-0), but a significant number of lncRNA genes were expressed at each stage. In both accessions the number of lncRNAs peaks at BCP stage and declines thereafter. In Col-0 there were 333 (UNM), 347 (BCP), 217 (TCP) and 139 (MPG) lncRNAs with expression values above 3 TPM and in Ler-0 270 (UNM), 301 (BCP), 174 (TCP) and 137 (MPG). The reduced numbers of lncRNAs expressed at later developmental stages resembles the trend for coding RNAs and distinct early and late lncRNA clusters were apparent (Fig. 2E).

In summary, the quantification of gene expression in developing pollen by RNA-seq analysis is in close accord with previous microarray analyses in Ler-0 (Honys and Twell 2004) but delivers new information about both protein coding and non-coding transcripts including data for different *Arabidopsis* accessions.

### Developmental transcriptome profiles reflect changes in numerous biological processes

**Fig. 3:**
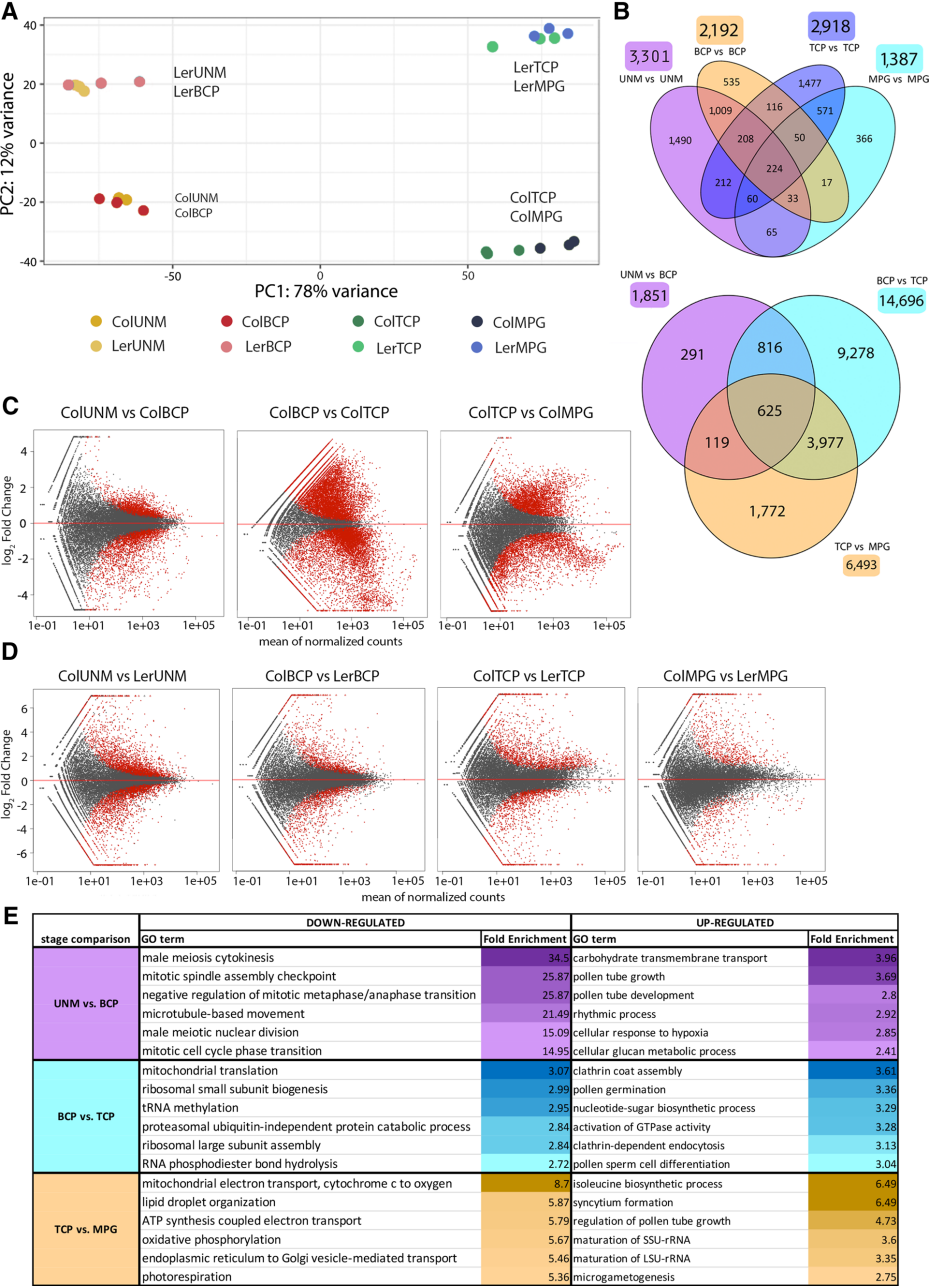
Differential gene expression throughout pollen development. **A** PCA plot of RNA-seq data. Four clusters separate early and late stages and distinguish Col-0 and Ler-0. **B** Venn diagram (upper panel) showing numbers of DEGs in pairwise comparisons of pollen stages of Col-0 and Ler-0. Venn diagram (below) of the total number of DEGs and the overlaps between stage transitions (Col-0). **C** MA plots of DEGs in stage transitions (Col-0). The most prominent changes occur during BCP¬-TCP transition. DEGs with padj < 0.05 are in red. **D** MA plots of DEseq2 results between accessions at each stage. The highest number of DEGs are at UNM stage between Col-0 and Ler-0. DEGs with padj < 0.05 are in red. **E**. Table of the six most enriched GO terms between stage transitions

Correlation analysis of gene expression profiles across developmental stages and between platforms (RNA-seq and microarray) unsurprisingly showed the highest similarity between adjacent early (UNM-BCP) and late (TCP-MPG) stages (Fig. 2A). Principal component analysis (PCA) of RNA-seq data identified four clusters, highlighting the greater similarity between accessions rather than between early and late pollen developmental stages (Fig. 3A). The similarity between early and late developmental stages was also apparent by hierarchical clustering, with two main branches according to accession (data not shown).

We investigated differential gene expression (DGE) with the DESeq2 package and set thresholds of statistical significance to log_2_ fold change < − 1 or > 1 and adjusted *p* value < 0.05. In Col-0, there were 1851 differentially expressed genes (DEGs) in the two early stages, with 547 up-regulated in UNM and 1304 up-regulated in BCP (Fig. 3B, D, Supplementary File 2). The major developmental shift was during BCP-TCP transition, resulting in 14,696 DEGs. While 8433 transcripts were upregulated in BCP, only 5763 were upregulated in TCP (Fig. 3D, Supplementary File 2), confirming the reduced transcriptome complexity after BCP stage (Honys and Twell 2004). In two late developmental stages, 6493 DEGs were identified, with 2915 transcripts upregulated in TCP and 3578 in MPG (Fig. 3D, Supplementary File 2). There was substantial overlap in DEGs between developmental stages, with 3977 DEGs shared between BCP-TCP and TCP-MPG transitions and 625 DEGs shared across all stage transitions (Fig. 3B). Conversely, the majority of DEGs between BCP and TCP stages were unique, further supporting the hypothesis of a major transition between these stages. Similar trends, namely the major developmental shift of 13,349 DEGs between BCP and TCP stages, were also observed in Ler-0.

The extent and dynamics of gene expression changes were also examined in individual developmental stages between accessions. The numbers of genes expressed at each stage were similar for the two accessions (Fig. 2D). The numbers of DEGs between accessions declined by 57.8% from UNM to MPG, again reflecting the general decrease in overall transcriptome complexity. Surprisingly, more DEGs were observed in TCP than in BCP. The highest number of DEGs between accessions was found at UNM stage (3301 genes), followed by 2918 and 2192 DEGs in TCP and BCP, respectively, with 1387 DEGs in MPG (Fig. 3B, C). The significant overlap between DEGs in UNM and BCP (1009 genes) and between MPG and TCP (571 genes) highlights the reduced variability of DEGs within early- and late-stage clusters (Fig. 3B).

Next, we explored the potential biological significance of transcriptome changes by examining developmental shifts in GO categories (Fig. 3E). During UNM-BCP transition, 115 positively enriched GO categories were upregulated in UNM. These included terms associated with mitotic events (cyclin dependent protein phosphorylation, phase transition control or spindle organization) indicating preparation of microspores for cell division. Notably, 17 of 41 plant kinesins are differentially expressed. Transcripts upregulated in BCP were mainly connected to glucan metabolism and transmembrane transport, including cell wall-associated transcripts. Remarkably, 22 transcripts associated with pollen tube development or pollen tube growth are also among upregulated DEGs. In more detail, 9 B-box type zinc finger and 20 EF hand domain proteins were among BCP upregulated DEGs with overrepresented protein domains. B-box transcription factors have diverse roles including flowering time regulation or stress tolerance and it has also been noted that they are expressed in pear pollen (Cao et al. 2017). The EF hand domain proteins include several calcium dependent protein kinases (CPK20, CPK16, CPK26, CPK6, CPK2), which continue to increase in expression to TCP stage and are reported to play a role during pollen tube growth (Yang et al. 2021).

A major transcriptomic shift was associated with BCP-TCP transition. GO analysis of the 13,349 (Ler-0) to 14,696 (Col-0) DEGs uncovered 325 enriched biological process terms. BCP stage was defined by upregulated transcripts connected to translation, from tRNA and ribosome biogenesis to mRNA maturation and protein folding. From TCP stage onwards, the enrichment of translation-related GO categories was drastically reduced, and transcripts connected to pollination, pollen germination and pollen tube growth increased. For example, 40 transcripts were connected to pollen germination and 99 to pollen tube development. BCP-TCP transition was also characterized by upregulation of transcripts connected to vesicle transport including both exocytosis and clathrin-dependent endocytosis, while the signalling terms activation of GTPase activity, Rab protein signalling and Ras protein signalling were also enriched along with glycerophospholipid metabolic process. For most of these terms, more than 50% of total genes belonging to the terms were differentially expressed, indicating a major transcriptome shift linked to these processes. The most enriched GO terms for each stage transition are summarized in Fig. 3E.

**Fig. 4:**
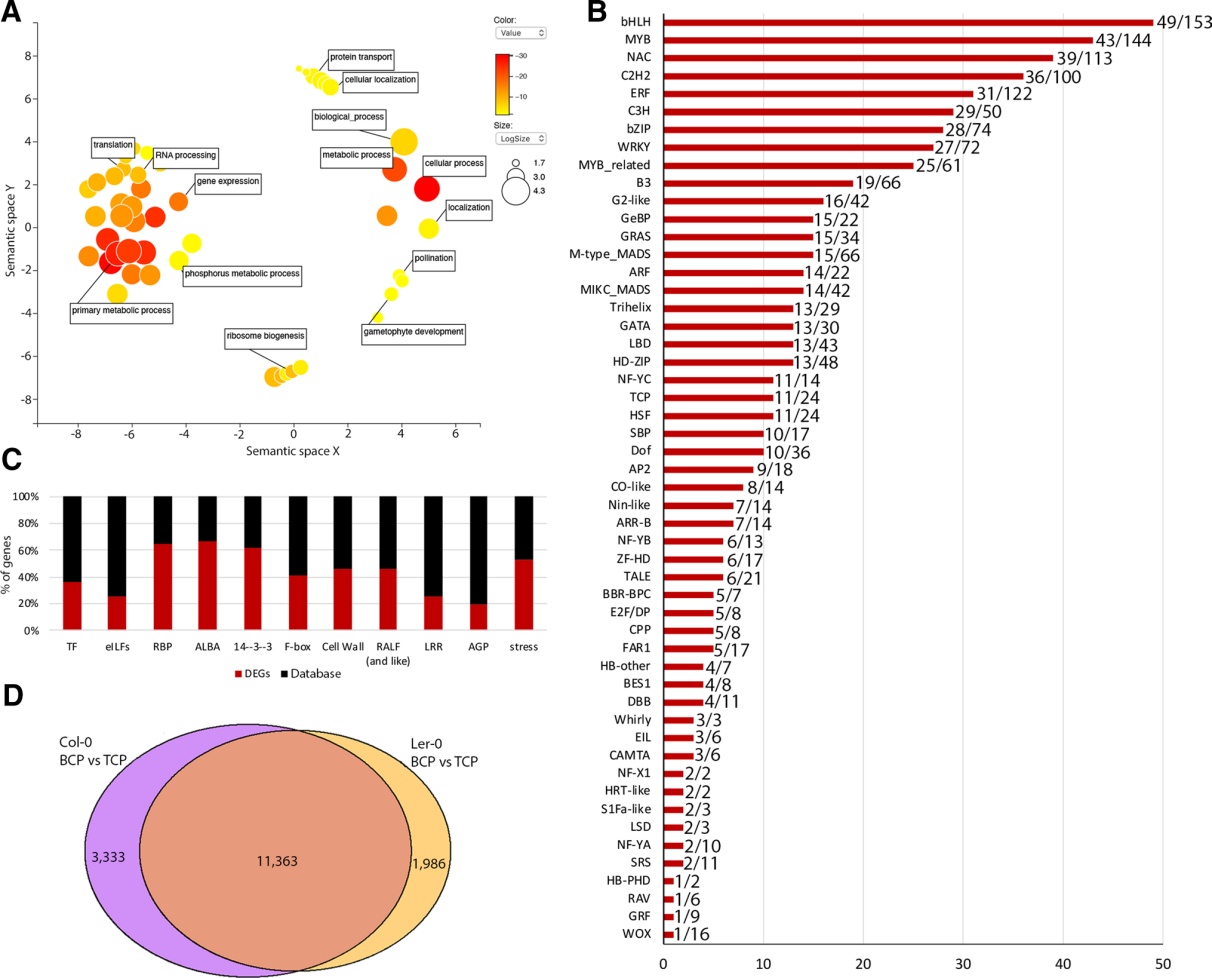
Functional categorization of differentially expressed genes in developing pollen. **A** ReviGO plot of enrichment analyses of BCP vs TCP DEGs indicating major transcript groups. **B** Numbers of DE TFs during BCP-TCP transition. Each bar shows how many DE TFs are represented out of the total. **C** Percentage of DEGs in selected groups compared to reported numbers. **D** Venn diagram showing overlap between BCP-TCP DEGs in Col-0 and Ler-0

Genes encoding regulatory proteins, involved in transcription, signalling cascades, protein modification and degradation were also modulated during BCP-TCP transition (Fig. 4A). Among transcription factors (TFs) 37% (626 genes from 52 TF families) showed altered expression, with 397 TFs upregulated in BCP and 229 in TCP (Fig. 4C, Supplementary File 3). Notable TF families, with at least 50% of members differentially expressed and a minimum of 75% of DE members upregulated in the BCP stage, were AP2, auxin response factors (ARF), BES1, CAMTA, CO-like, GeBP, homeobox-other (HB-other) and homeobox-PHD finger (HB-PHD). Other TF families, represented by less than 50% of members among DEGs and mainly upregulated in BCP, were B3, basic/helix-loop-helix (bHLH), GATA and homeodomain-zip (HD-zip). TFs upregulated at TCP stage belonged to EIL, NF-X1 and S1Fa-like gene families. Several large TF families, including basic leucine zipper (bZIP), C2H2, ethylene responsive factors (ERF), MYB and MYB-related, C3H, NAM, ATAF, and CUC (NAC), WRKY or MADS-box showed similar numbers of up- and down-regulated members at each stage, suggesting that their activity is modulated by exchange among members during BCP-TCP transition.

**Fig. 5:**
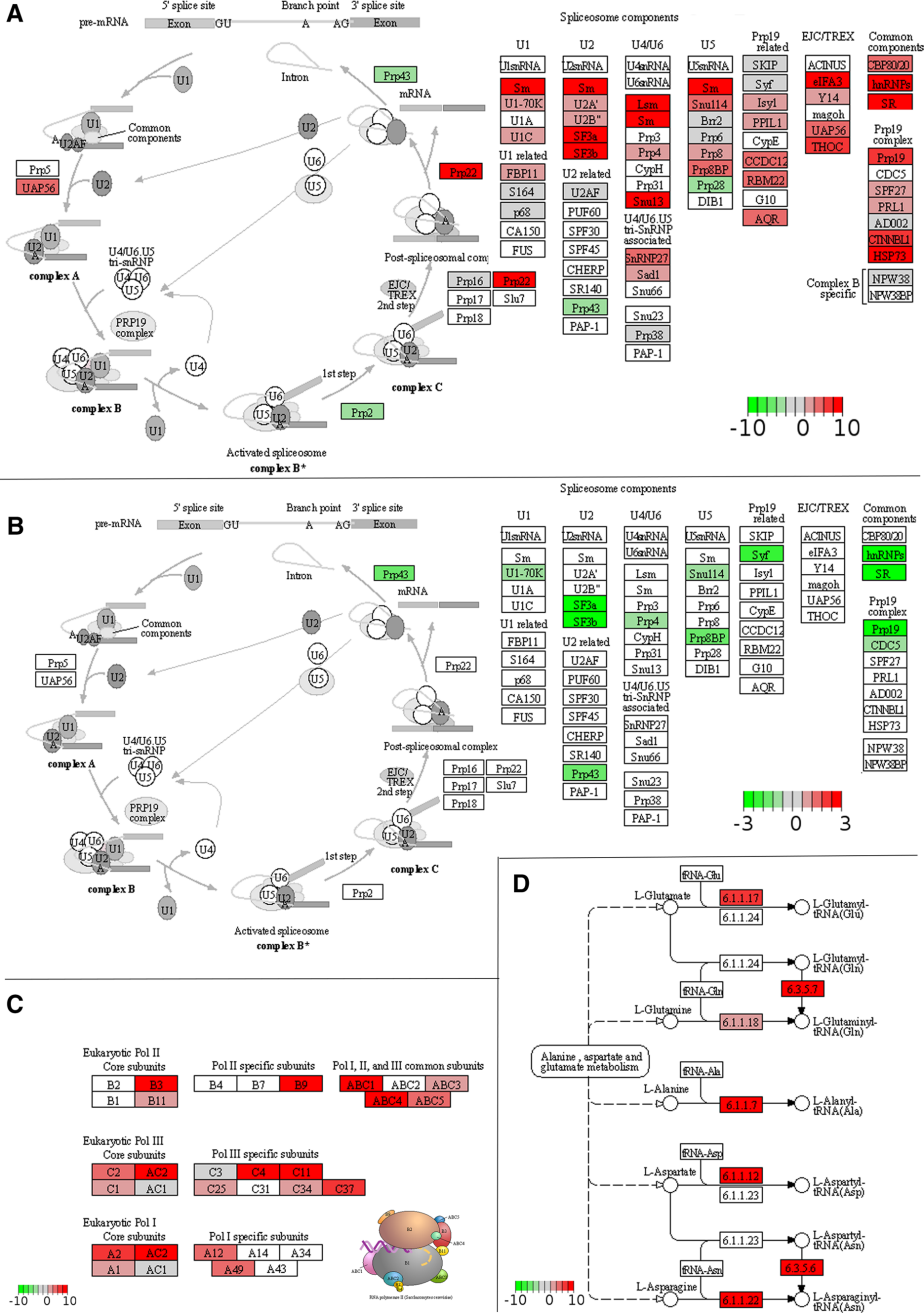
KEGG pathway enrichment during BCP-TCP stage transition. KEGG pathway analysis shows upregulation of mRNA processing (**A** spliceosome, transcripts), transcription (**C** RNA polymerase) and translation (**D** tRNA synthesis) pathways in BCP compared to TCP. Spliceosome genes show peak expression in BCP, but protein expression peaks in TCP (**B** spliceosome, proteins). Plots were rendered by Pathview. Genes upregulated in TCP (green) or BCP (red) are highlighted. The scale indicates log_2_fold change of DEGs

We further examined the expression dynamics of specific groups of genes. More than 60% (756 genes) of genes encoding RNA-binding proteins (RBP) were differentially expressed during BCP-TCP transition (Fig. 4C). Most RBPs were highly expressed during early stages, with 568 genes upregulated in BCP and only 190 with higher expression in TCP. Notable genes downregulated in TCP included four ALBA (acetylation-lowers binding affinity) superfamily protein genes (Náprstková et al. 2021) and seven encoding all subunits of nascent polypeptide-associated complex (Fíla et al. 2020). Other markedly shifted groups included stress-associated transcripts, cell wall transcripts, 14-3-3 proteins, F-box proteins or RALFs (rapid alkalization-like factors), RALF-like groups and receptor kinases, as well as translation initiation factors (eIFs; Fig. 4C). Accordingly, enriched KEGG pathways highlighted enrichment of processes related to mRNA metabolism, with most of the transcripts being upregulated in BCP (Fig. 5A, B, C). The large overlap in DEGs (11,306) between accessions highlights the conservation of developmental regulation during BCP-TCP transition in *Arabidopsis* (Fig. 4D).

The final developmental phase from TCP to MPG shows fewer changes compared with BCP-TCP transition and is enriched for 379 GO terms. The 2915 DEGs upregulated in TCP are enriched for energy and biosynthetic processes including ATP synthesis coupled proton transport and electron transport chain. The expression of these gradually declines from UNM to MPG, similar to those coding for vesicle transport- and exocytosis-related proteins. Transcripts connected to pollen, pollen tube germination and pollen development (109 DEGs) are upregulated in TCP, while 36 DEGs upregulated in MPG are involved in pollen tube growth regulation and microgametogenesis. Notably, some MPG-upregulated transcripts are highly abundant with mean expression of 553 TPM, whereas average expression in MPG is only 29 TPM. A further 82 transcripts were included in the enriched term response to cold and 90 in response to abscisic acid. Of the 3,087 annotated DEGs upregulated in MPG, 26% (816 genes) are linked to stress response. In comparison with other stages, the upregulation of stress response transcripts is apparent. The list includes groups of genes responsive to salt, radiation, heat, cold, desiccation and chemical stimuli, as well as genes responsible for DNA repair, protein oxidation, signalling, transport and response to biotic stress.

Although translation-associated GO terms were also enriched, the overall expression of such transcripts was low when compared to similarly associated transcripts upregulated in earlier stages. For example, ribosome biogenesis is enriched in both BCP and MPG relative to TCP, but the mean expression of 127 genes in this category is 159 TPM in BCP and only 18.1 TPM in MPG. Therefore, a decline in the abundance of translation-related genes is apparent from BCP onward. Among GO terms shared between BCP and MPG, only four genes had higher expression in MPG than in BCP. These include ribosome biogenesis genes At3g22510 and REI1-LIKE1 (At4g31420; Cheong et al. 2021) and genes with translation functions, SUI1-family initiation factor (At1g54290; Bach-Pages et al. 2020) and TMA7 (At3g16040; Fleischer et al. 2006).

MPG-upregulated transcripts also showed enrichment for the cellular location of their predicted products, with for example 67 apoplast, 109 cell wall and 521 plasma membrane proteins. The complete set of enriched GO terms for all stages is plotted in the Supplementary Fig. 1.

In summary, transcriptome dynamics of developing pollen impacts numerous biological processes with major changes during phase transition from early to late stages. Early stages are accompanied by enhanced transcription of genes encoding proteins involved in translation, mRNA, and protein processing and also in cell division. Late phase dynamics suggest preparation for pollen desiccation by enhanced transcription of stress-related genes, and later for pollen activation and pollen tube growth, by accumulation and storage of transcripts associated with vesicular transport, energy metabolism or pollen tube growth.

### There are no major transcriptome changes between Col-0 and Ler-0 during pollen development

**Fig. 6:**
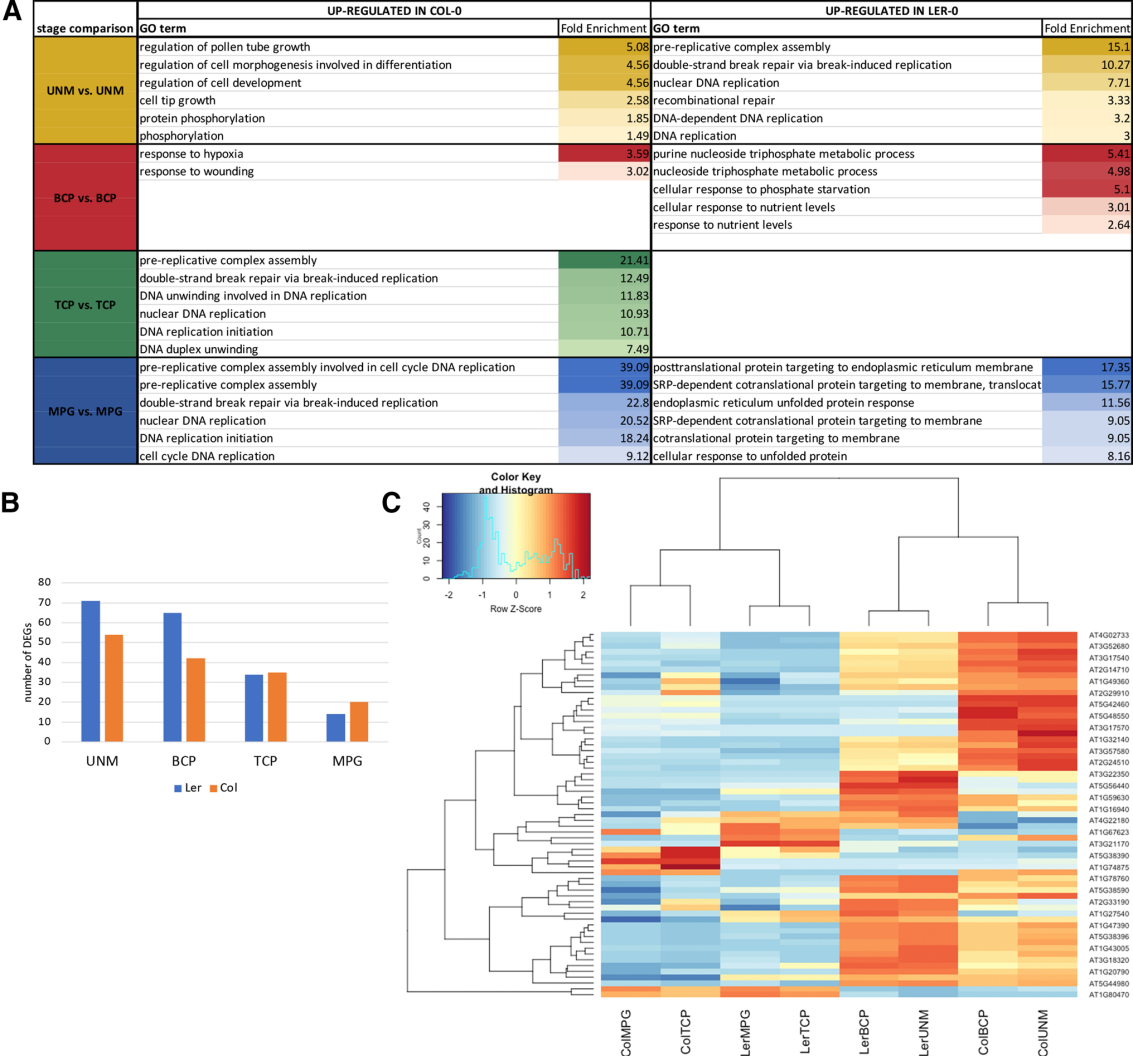
Functional categorization of differentially expressed genes between Col-0 and Ler-0. **A** Summary of the most enriched GO terms (≤ 6) among DEGs between Ler-0 and Col-0 sorted by stage. **B** Number of F-box genes differentially expressed between Ler-0 and Col-0 at each stage. **C** Heatmap of candidate F-box gene expression. There are similar trends between early and late developmental phases for most of the genes in both accessions

The analysis of developmental transcriptomes between Col-0 and Ler-0 accessions did not identify major differences in biological processes or metabolic pathways. Among processes with the highest fold-enrichment were GO terms related to DNA replication, but the associated transcripts were usually of low abundance (mean of 9.6 TPM in Col-0 and 2.2 TPM in Ler-0). The 13 enriched processes upregulated at UNM stage in Ler-0 included 6 DNA replication-associated terms, whereas transcripts representing replication connected GO terms were more abundant in Col-0 at TCP and MPG stages (Fig. 6A). Phosphorylation-associated transcripts as well as 10 transcripts related to regulation of pollen tube growth were also enriched in Col-0 at UNM stage. At MPG stage, Col-0 showed 51 enriched processes, whereas Ler-0 was enriched for transcripts involved in protein targeting to endoplasmic reticulum and the ubiquitin-dependant ERAD pathway.

Studies of in vitro pollen germination and tube growth have highlighted differences between *Arabidopsis* accessions. The carbohydrate type and concentration (Hirsche et al. 2017), pH (Rodriguez-Enriquez et al. 2013) and salt conditions (Azarov et al. 1990) of in vitro pollen germination media as well incubation temperature (Boavida and McCormick 2007) can lead to drastic differences in germination responses between Col-0 and Ler-0. The differential expression of sucrose-H + symporters are implicated (Sauer et al. 2004), specifically *AtSUC1* (*ARABIDOPSIS THALIANA SUCROSE-PROTON SYMPORTER 1*), which has reduced immunodetectable protein in Ler-0 pollen (Feuerstein et al. 2010). Similarly, we observed a log_2_fold difference of − 4.4 for *AtSUC1* (At1g71880) at UNM stage and reduced signal in Ler-0 throughout development (mean expression 66.3 TPM in Col-0 and 24.44 TPM in Ler-0).

Genes encoding F-box proteins were the most abundantly represented group of DEGs between accessions, representing 2.5% to 5% of DEGs, with the greatest variability at early developmental stages (Fig. 6B). Further, the mean (217 TPM) and median (120 TPM) values were non-negligible with maximal expression of a DE F-Box transcript (At4g27050) reaching 2010 TPM in UNM. The expression statistics and AGIs of F-box genes at each stage are given in Supplementary File 4. Protein ubiquitination was connected to 35 differentially expressed F-box proteins. Notably, five F-box genes were exclusively expressed in Col-0 (At2g04810, At3g47130, At5g38391, At5g42460, At5g56380), while two were only expressed in Ler-0 (At5g56440, At3g21170). Although some F-box transcripts differ in expression between accessions, their developmental expression profiles were similar for most of the genes (Fig. 6C).

### Pollen development is accompanied by isoform switches and differential exon usage

**Fig. 7:**
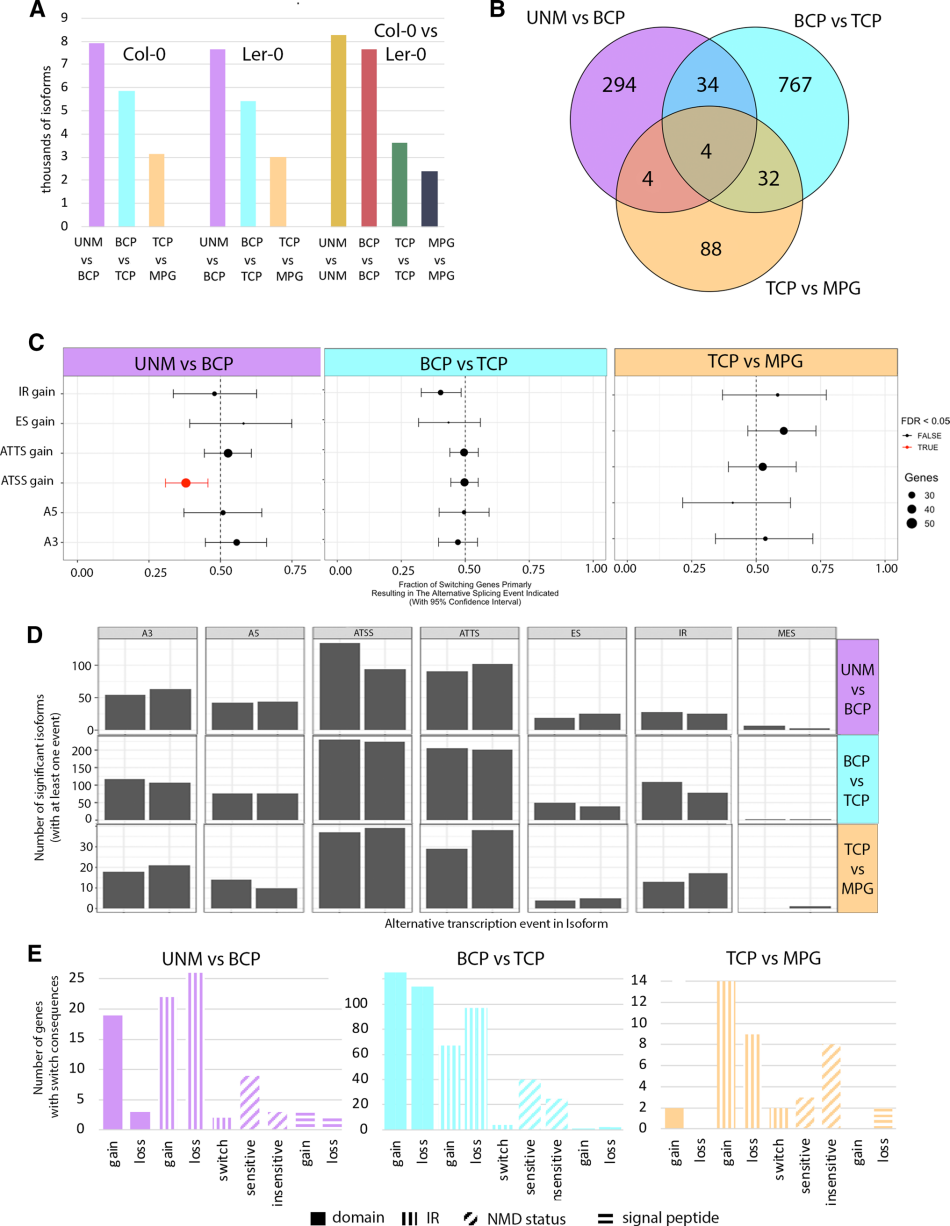
Analysis of differential isoform usage (DIU) in Col-0 pollen development. **A** The number of isoforms passing statistical threshold (both gene and isoform expression > 3 TPM) declines with stage progression. **B** Venn diagram showing high DIU variability with only 4 isoforms switches in all 3 pairs of stages compared. **C** Enrichment of alternative isoform types during Col-0 stage transitions. Only ATSS gain during UNM-BCP transition is significantly enriched. **D** The distribution of alternative isoform types during Col-0 stage transitions is conserved during pollen development. ATSS and ATTS events are always most frequent. **E** Switch consequences of DIU. IR is most frequent during UNM-BCP and TCP-MPG transitions. Change of domain presence is the most frequent isoform switch consequence during BCP-TCP transition

We used complementary methods to examine differential exon usage (DEU) across pollen developmental stages. First, we employed DexSeq using the scaled expression (TPM) of each isoform from RSEM analysis as input. Next, differential isoform usage (DIU) and its consequences were analysed using IsoformSwitchAnalyzeR with the same data input. In total, 1769 exons in 1132 genes showed significant DEU in Col-0 UNM-BCP transition. We detected DEU for 1769 exons in 1131 genes during BCP-TCP transition and 1037 exons in 588 genes for TCP-MPG transition. As expected, DIU events were much less common than DEU. DIU analysis identified 336 isoform switches in 204 genes during UNM-BCP transition, only 17 of which were DEGs. There were 837 isoform switches in 458 genes between BCP and TCP and 129 isoform switches in 78 genes during TCP-MPG transition (Supplementary File 5). We observed a similar trend in the number of expressed isoforms to the number of expressed genes, which decreased from UNM to MPG (Fig. 7A). Only four transcript isoforms switched between all three stage transitions, with most being stage-specific events (Fig. 7B).

The isoform switches were further categorized as intron retention (IR), exon skipping (ES), alternative transcription start sites (ATSS), alternative transcription termination sites (ATTS), alternative 5’ splice site (A5) and alternative 3’ splice site (A3), multiple exon skipping (MES) or mutually exclusive exons (MEE). The distribution of alternative transcription events was comparable for all three stage transitions, with alternative transcription start sites and alternative transcription termination sites being the most represented followed by alternative 3’ splice site and intron retention. Only a few isoforms switches resulted in multiple exon skipping (Fig. 7C, D). We analysed the consequences of isoform switches focussing on changes in coding potential, domain and signal peptide presence, intron retention or sensitivity to nonsense-mediated decay (NMD), that may drastically affect protein cellular localization or function. There were 90 switch consequences for 69 genes during UNM-BCP transition. This resulted in domain changes in 20 genes (18 gain, 2 loss), while 50 switches involved intron retention and five presence/absence of a signal peptide. Changes related to potential sensitivity to NMD, which was integrated from the presence of premature termination codons in isoform’s sequence, was observed for 12 genes. In case of 3 gene, the NMD sensitivity led to decreased expression of the sensitive isoform. Similar results were observed throughout pollen development with the majority of DIUs involving intron retention (UNM-BCP and BCP-TCP) or resulting in a change in domain presence (TCP-MPG) (Fig. 7E).

GO enrichment for biological function was calculated based on the annotation of genes with DEU and DIU and the most significant genes and those with switch consequences were investigated manually. The comparison of DEU and DIU for UNM-BCP transition resulted in the enrichment of genes involved in meiosis cytokinesis together with chromosome organization. DEU occurred in transcripts connected to cytoskeleton and vesicle trafficking (including vesicle transport along actin filament, cytoskeleton organization or vacuole transport). Involvement of developmental genes was represented by the terms pollen development and microgametogenesis. There were 11 genes with DIU involved in posttranscriptional regulation of gene expression and 12 genes with a role in mRNA processing including splicing factors and ribonucleases.

**Fig. 8:**
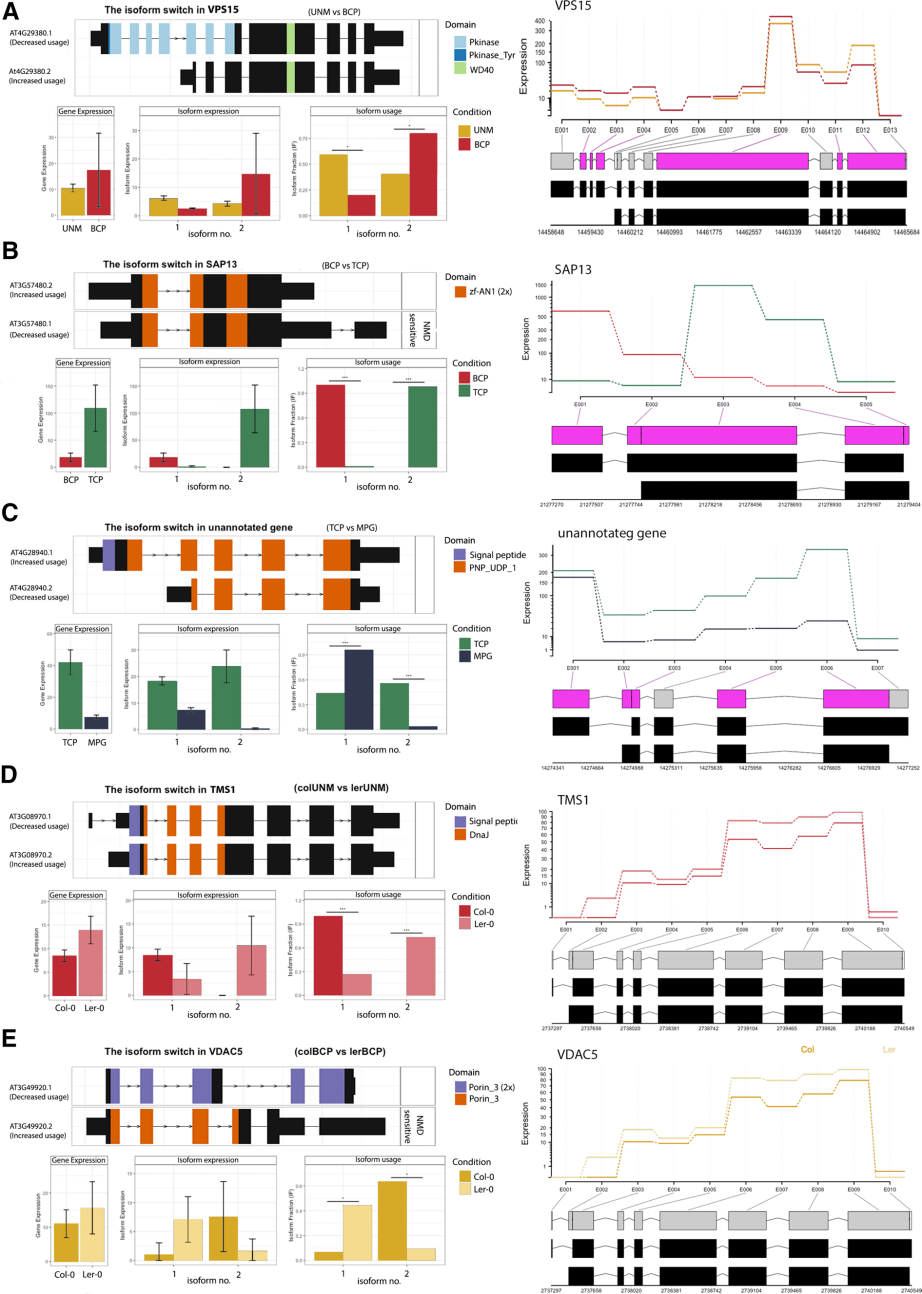
Visualization of differential isoform usage (DIU) and differential exon usage (DEU) for selected transcripts. Isoform switches in selected genes are shown on the left, while difference in exon usage is shown on the right. The line plot represents exon usages for each condition (developmental stage), the lower part indicates significantly differentially expressed exons by pink colouring. **A** VPS15 isoform 2 lacking the kinase domain is dominant after UNM-BCP transition. **B** SAP13 isoforms switch during BCP-TCP transition. **C** AT4G28940.1 (unannotated) has a signal peptide and is only expressed in MPG. **D** ERDJ3A isoform 2 is only present in BCP of Ler-0. **E** A different VDAC5 isoform in BCP stage of each accession results in up-regulation of an isoform missing one of the porin domains and is NMD sensitive in Ler-0 DIU statistics and domain presence are shown on the left

Genes with switch consequences resulting in domain changes included VPS15 kinase (Xu et al. 2011; Liu et al. 2020), which is involved in autophagy (Fig. 8A). Interestingly, the kinase domain was lost from the transcript in BCP. Another candidate was the H^+^ATPase AHA3 (At5g57350; Bock et al. 2006; Robertson et al. 2004) with the isoform lacking ATPase domains dominant in BCP. Other candidates emerged when isoforms with high difference in isoform fraction (dIF) were examined. *THERMOSENSITIVE MALE STERILE 1* (*TMS1*) isoform 2 (At3g08970.2; Yang et al. 2009; Ma et al. 2015) was only expressed in BCP, whereas isoform 1 was dominant in UNM. Similarly, histone deacetylase HDA5 (At5g61060), a salt stress-responsive protein also involved in flowering time regulation (Luo et al. 2015; Ueda et al. 2017), switched from isoform 1 to 2 during UNM-BCP transition.

During BCP-TCP transition in Col-0 there were changes in 836 isoforms of 458 genes and for 331 genes these switches had consequences. In total 67 isoform switches resulted in change in NMD sensitivity of which 27 NMD sensitive isoforms were downregulated. In Ler-0, 145 switch consequences were identified among the 548 isoforms of 304 genes. In total, 134 genes showed DIU in both accessions. Similar to the DGE analyses, the BCP-TCP transition accounts for the greatest number of isoform switches. The GO analysis of the genes with DIU showed enrichment in GTPase activity, membrane docking, mRNA splicing, protein transport and vesicle trafficking. Two most significant isoform switches resulted in the change of sensitivity to NMD via the dominance of the NMD-sensitive isoform of stress-responsive gene SAP13 (At3g57480, Zinc finger AN1 domain-containing stress-associated protein 13, Fig. 8B; Dixit et al. 2018) and of RPA2A (At2g24490, Replication protein A component responsible for transcriptional gene silencing; Aklilu et al. 2020; Xia et al. 2006). In AGAMOUS-LIKE MADS-box protein AGL65 (Adamczyk and Fernandez 2009) the BCP-prevailing isoform At1g18750.5 lacks the MADS-box domain, which is present in one of the most expressed isoforms (At1g18750.4) in TCP.

The TCP-MPG included 129 isoform switches with 30 causing the switch consequences. For example, 11 switches resulted in the change in NMD sensitivity and 8 NMD sensitive isoforms became downregulated during the TCP-MPG transition. Functionally, the progression is characterized by differential usage of isoforms enriched for transporters (17 in total). Examples include the functionally important PHOSPHOLIPASE A2 DELTA (At4g29470) which is essential for pollen germination and pollen tube growth (Kim et al. 2011) and the pollen-specific mechano-sensitive channel-like protein MSL8 (At2g17010) which has a protective role during pollen hydration (Hamilton et al. 2015). Other candidates with switch consequences include the shorter TCP dominant isoform 2 of phosphorylase At4g28940, which lacks a signal peptide (Fig. 8C), while an isoform switch in Glyoxalase GLXI-LIKE9 (At2g32090; Schmitz et al. 2018) results in the gain of the glyoxalase domains.

We found more differences in alternative splicing (AS) between Col-0 and Ler-0 during early development. There were 560 isoform switches in 330 genes in UNM and 650 in 375 genes in BCP, of which 150 and 180 had consequences respectively. In late development, there were 289 switches in 153 genes in TCP and 216 switches in 117 genes in MPG. These isoform switches had consequences in 78 and 64 genes, respectively. Isoform switches between accessions were detected in 16 genes across all four stages. These data might reflect general differences in pollen isoform usage between accessions particularly in early developmental stages. In most cases, switches were connected to a specific developmental stage. One of the most significant switches in UNM resulted in a change of isoform usage for heat shock protein THERMOSENSITIVE MALE STERILE 1 (Yang et al. 2009; Fig. 8D). Genes with isoform switches in BCP were enriched for RNA binding proteins and biological processes including translation and RNA processing. These categories included seven 60S and three 40S ribosomal proteins and eukaryotic translation initiation factor 3 subunits C and D. Another interesting candidate was the voltage dependent mitochondrial channel VDAC5 (At3g49920; Fig. 8E), which shows polymorphisms associated with flowering adaptation (Tabas-Madrid et al. 2018).

### Proteomic analyses of pollen development

**Fig. 9:**
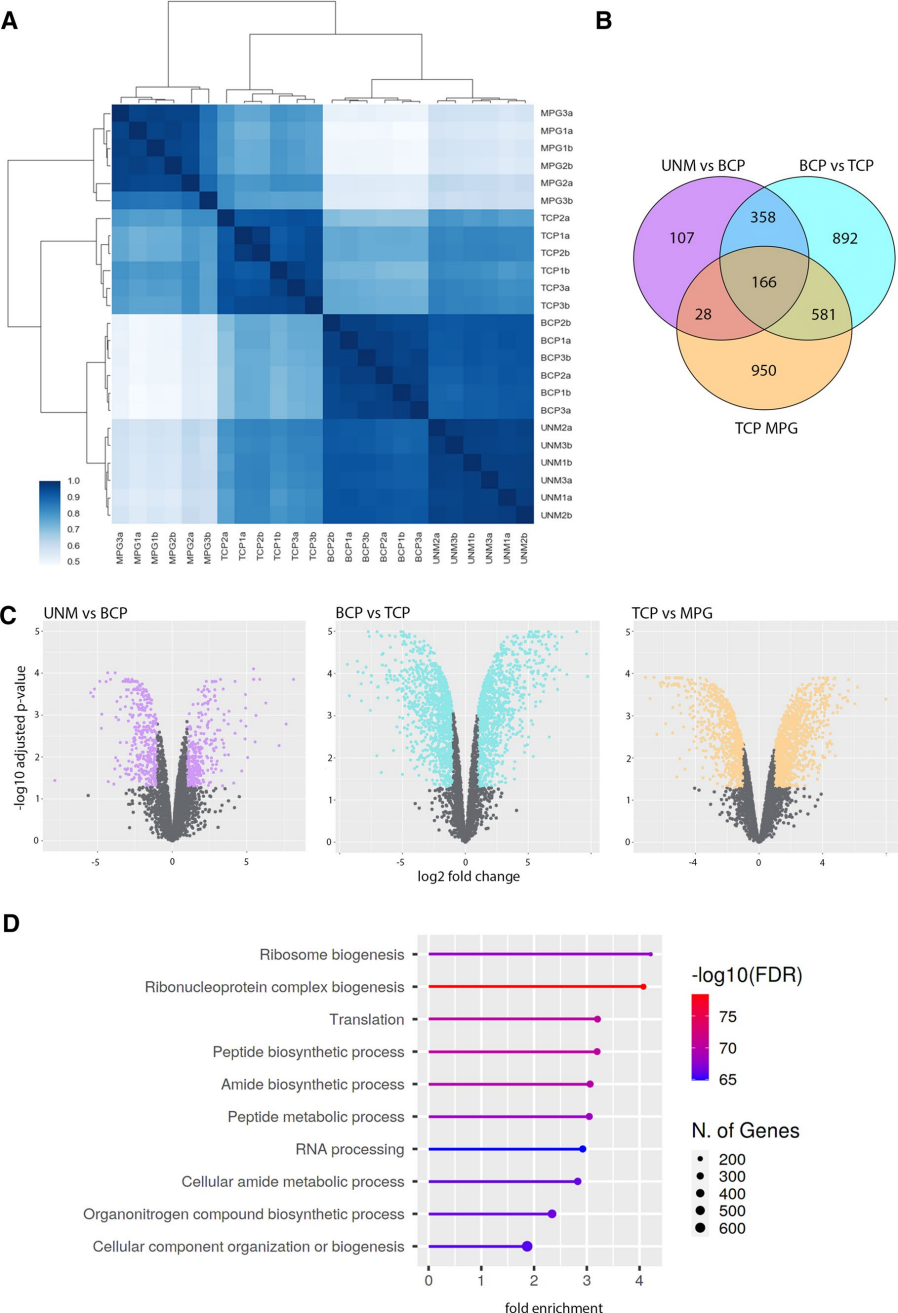
Differential protein expression during pollen developmental stage transitions. **A** Pearson correlation of biological and technical sample replicates used in this study. **B** Venn diagram of DEPs. Most abundance differences are stage transition-specific; 166 proteins change their expression during each transition and 581 proteins are differentially expressed between BCP-TCP and TCP-MPG. **C** Volcano plots of expressed proteins in pairwise comparisons of UNM-BCP, BCP-TCP and TCP-MPG. The highest number of DEPs occur during BCP-TCP transition. **D** The 10 most enriched GO terms for DEPs during BCP-TCP transition highlights an increase in translation related proteins

Samples of four pollen developmental stages of Col-0 were processed and analysed by LC–MS/MS, with each stage represented by three biological replicates and two technical replicates. In summary, we identified 4965 protein groups (PGs) in the whole data set (Supplementary file 6). The highest numbers of PGs were identified in TCP (3197 PGs, PGs detected in four of six analyses per sample type using at least two peptides) and in MPG (3100 PGs), while the lowest number of PGs was detected in BCP (2716 PGs). In UNM samples, we identified 2963 PGs. Pearson correlation of the total of 6 replicates for each stage shows highest similarity between early stages (UNM and BCP) and a good correlation between replicates (Fig. 9A).

Differentially expressed proteins (DEPs) between adjacent stages were identified. The threshold for significant quantitative change was set as log_2_ fold change (FC) < − 1 or FC > 1, and adjusted *p* value < 0.05 (Supplementary File 7). Proteins with quantified replicates in only one sample of the two and zero in other were retained as qualitative changes. During UNM-BCP transition, 312 and 347 proteins were quantitatively upregulated, while 232 and 49 proteins were qualitatively upregulated in UNM and BCP, respectively (Fig. 9B, C). Both quantitative and qualitative changes were analysed together for functional consequences. The transition from UNM to BCP resulted in higher abundance of proteins involved in lipid storage, retrograde vesicular transport, and cell-wall organization. These groups included glycine-rich proteins, oleosins, expansins, and the cell wall regulators pectinesterases and pectinesterase inhibitors. Also, three subunits of the coatomer complex were up-regulated, epsilon-1 (At1g30630), zeta-2 (At3g09800) and zeta-3 (At4g08520) subunits (Cabada Gomez et al. 2020). Numerous proteins with a role in pollen germination and pollen tube growth showed enrichment. For example, three pollen coat proteins, extracellular lipase 4 (At1g75910; Updegraff et al. 2009), extracellular lipase 6 (At1g75930; Dong et al. 2016) and glycine rich protein 17 (At5g07530), and five pollen tube tip proteins including pectinesterase inhibitor 1 (At1g48020; Röckel et al. 2008), LOST IN POLLEN TUBE GUIDANCE 2 (At3g02810) *73*), ANXUR1 (At3g04690; Boisson-Dernier et al. 2009), pollen receptor like kinase 6 (At5g20690) and ATP-binding cassette G28 (At5g60740).

The largest number of DEPs were identified for the BCP-TCP transition with 1,048 and 946 proteins with quantitative up-regulation in TCP and BCP respectively (Fig. 9B, C, Supplementary File 7). There were 652 qualitatively upregulated proteins in TCP and 165 in BCP. Among BCP upregulated proteins, there was an enrichment in proteins involved in cell wall modification mainly consisting of pectinesterases (PME4, PME5, PPME1, PME67, PME49 and PME48) and pectinesterase inhibitors (PME43, PME28 and VGDH2). A further group of proteins containing the c11 pectin lyase fold domain represented connection to carbohydrate metabolism. Another enriched term was lipid storage, which was represented by four oleosins and one glycine-rich protein. Pollen tube growth regulating proteins also showed high fold enrichment as well as oxidation–reduction process proteins. Numerous processes connected to saccharide metabolism and utilization were enriched in BCP including sugar transport, pyruvate, pectin, sucrose, galactose, glutamate, fructose, malate, UDP-rhamnose metabolism as well as processes involved in gluconeogenesis as aspartate and glycerol metabolic processes. Biosynthesis of nucleotides is also represented.

At the beginning of the late developmental phase, proteins connected to mRNA processing, translation and protein modification were upregulated (Fig. 9D). For example, enriched processes included rescue of stalled ribosome, ribosome subunit assembly, regulation of translation, protein maturation, folding and targeting. There were 93 proteins possessing an RNA recognition motif, 15 proteins annotated as tRNA binding and 134 ribosomal proteins. Spliceosome was also observed as an enriched KEGG pathway (Fig. 5B). The BCP and TCP transcriptomes and corresponding proteomes overlapped for several spliceosome components including the PRP19 complex, which showed higher transcript abundance in BCP. Spliceosomal protein expression was then upregulated during BCP-TCP transition, suggesting uncoupling of transcription and translation between early and late stages (compare Fig. 5A and B). Also, we observed enrichment of proteins involved in posttranscriptional regulation of gene expression and negative regulation of transcription, which includes five histone deacetylases, the chromatin regulator ZUOTIN-RELATED FACTOR 1 (ZRF1) and Argonaute 4 (AGO4).

During the TCP-MPG transition 712 proteins were quantitatively upregulated in MPG, while 1011 were more abundant in TCP. A further 283 and 205 proteins were qualitatively upregulated in TCP and MPG respectively (Fig. 9 B, C). The DEPs upregulated in TCP included the dominant categories connected to metabolism of fatty acids, nucleotides, and carbohydrates together with vesicle-mediated transport. Among DEPs upregulated in MPG, there are proteins responsible for epigenetic post-transcriptional silencing, namely ARGONAUTE 1 (AGO1), AGO5, AGO9, SET DOMAIN PROTEIN 18 (SUVR2) or NUCLEAR RNA POLYMERASE D1B (NRPD1B). The enrichment of processes connected to chromatin remodelling, chromosome organization, gene silencing and siRNA production indicate epigenetic changes taking place during pollen maturation. The quantitative changes include proteins mainly connected to translation and protein metabolism together with mRNA transport, processing, and ribosome assembly. Interestingly, photosynthesis and plastid translation are among the upregulated protein groups. Quantitative changes included stress response proteins including 32 cold stress, 25 heat stress, 11 virus response and 42 defence response proteins. In general, the MPG proteome is characterized by expression of stress-related proteins and those associated with post-transcriptional and epigenetic regulation of transcription. In summary, early development is characterized by the greater abundance of proteins required for pollen development, and transport and metabolism, than in the later phase, which is followed by up-regulation of mRNA processing, translation regulation, stress proteins and epigenetic regulators.

Comparison of our proteomic dataset with a reference data set of 3491 proteins identified in mature *Arabidopsis* pollen (Grobei et al. 2009) revealed 2791 (79.9%) protein groups in common. Among these, 2345 proteins (83.9%) were detected in mature pollen (riBAQ > 1) with an average riBAQ value of 328.

### Major gene groups for active translation and mRNA storage emerge from the analysis of developmental transcriptomic and proteomic data

**Fig. 10:**
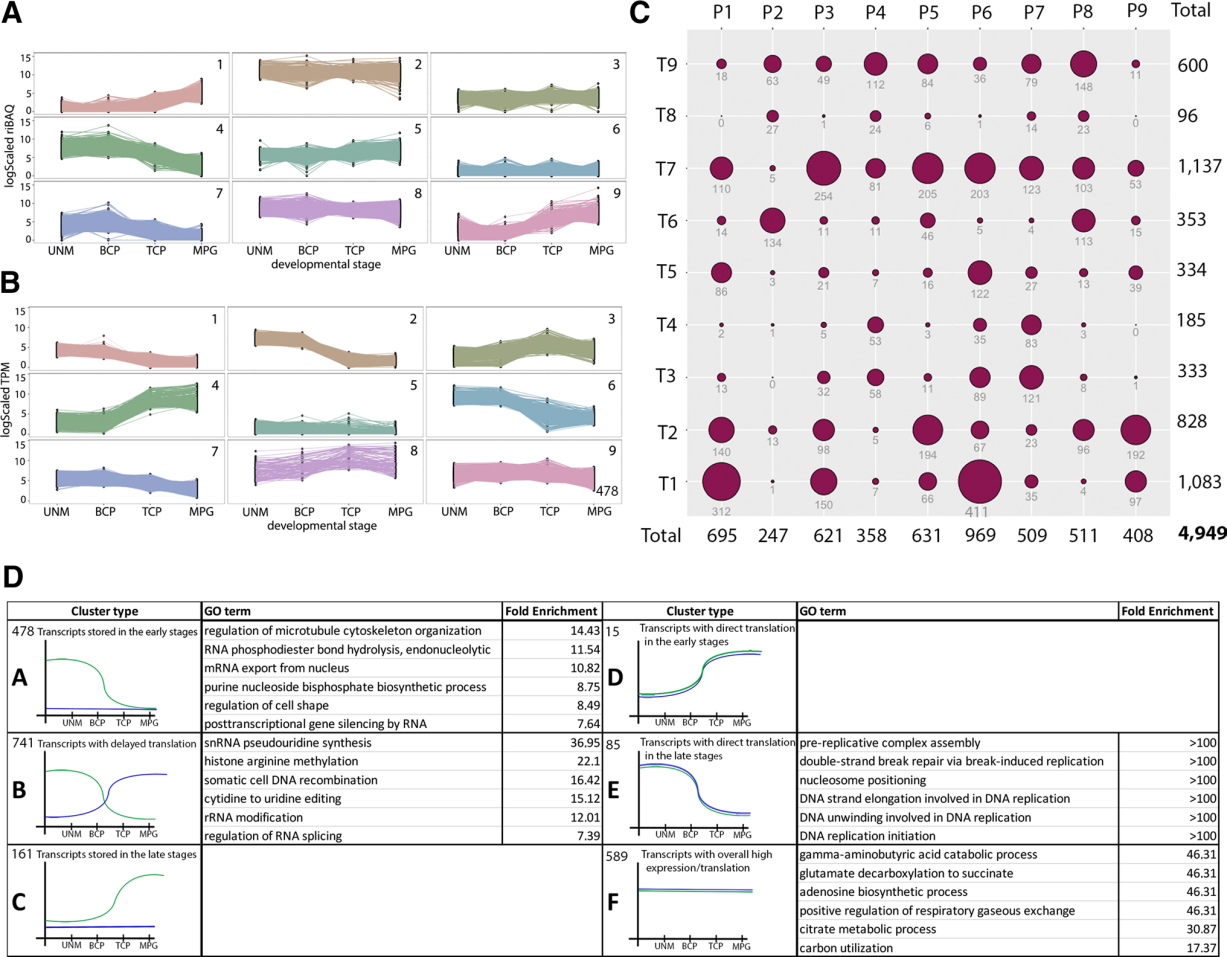
Integration of transcriptomic and proteomic data in developing Col-0 pollen. **A** k-means clustering of expression of 4,949 proteins. **B** k-means clustering of expression for 4,949 transcripts. **C** Matrix indicating numbers of overlapping genes in transcripts and protein clusters. **D** Transcript clusters distinguished according to RNA and protein profiles. Plots indicate relative transcript (green) and protein (blue) expression for each cluster. The numbers of transcripts in each cluster and the six most enriched GO terms and their fold enrichment are listed

To explore the fate of transcripts expressed in developing pollen, we compared transcriptomic and proteomic data in Col-0. There were 4949 shared genes between datasets, and we focussed our analyses on these (Supplementary File 8). k-means clustering of log_2_ scaled TPM (transcripts) and log_2_ scaled riBAQ values (proteins) resulted in nine clusters covering possible relationships of expression profiles in both datasets (Fig. 10A, B, Supplementary File 9). The majority (61.6%) of transcripts were present in three clusters (T1; 1,083 genes; T2, 828 genes; T7, 1,137 genes) that declined in abundance during development, while the least represented clusters (T4, 185 genes; T8, 96 genes), which comprised only 5.7% of all transcripts, increased in abundance at late stages of pollen development.

The top three proteome clusters (P1, P5, P6) comprised almost half (46%, 2295 proteins) of all proteins. These either increased in abundance in later stages (P1, 695 proteins), or remained relatively stable (P5, 631 proteins; P6, 969 proteins). The three least represented clusters (P2, P4, P9), which comprised 20.5% of all proteins also showed contrasting profiles, although protein distribution among them was more uniform. P2 (247 proteins) showed abundant and stable expression, P4 (358 proteins) declining in late developmental stages, while P9 (408 proteins) increased in later stages, like P1 (Fig. 10B).

To determine the relationship between transcript and protein cluster pairs, we constructed an overlap matrix (Fig. 10C). To describe the fate of different transcript groups, we selected six patterns of transcriptomic/proteomic overlap that were named A to F (Fig. 10D, Supplementary File 10). Group A includes transcripts present in UNM and BCP, but with limited translation throughout pollen development (Fig. 10D (A)). Group B highlights transcripts with peak expression in early stages but maximum protein accumulation in TCP and MPG, indicating delayed translation (Fig. 10D (B)). Group C comprises transcripts accumulating mostly in later stages and showing limited translation (Fig. 10D (C)). These may include stored transcripts that are translated during pollen germination and tube growth. Groups D and E represent transcripts that are directly translated at early or late developmental stages, respectively (Fig. 10D (D, E)). Group F reflects transcripts with stable, stage-independent expression and direct translation (Fig. 10D (F)). Together, these patterns of overlap illustrate different possible modes of transcript usage.

We examined the biological and molecular processes enriched in each group (Fig. 10D). Group A was enriched for GO categories related to cytoskeleton organization. Other enriched categories included RNA metabolism and processing and posttranscriptional gene silencing by RNA. In addition to mRNA and cell wall connected genes, transcripts involved in meiotic cell cycle and mitotic cell cycle were also enriched in group A.

In Group B, transcripts from other GO categories connected to RNA processing were present, as well as those associated with ribosome biogenesis and translation, with exemplary terms: positive regulation of mRNA splicing via spliceosome, ribosomal small/large subunit biogenesis or protein folding, as well as posttranscriptional gene silencing’. KEGG pathway analysis also showed enrichment for RNA-associated pathways. The connection of group B transcripts to RNA processing is further highlighted by enrichment for proteins containing RNA recognition motifs and/or WD domains.

Group C transcripts did not show enriched biological processes, but eight genes were connected to pollination and pollen tube growth, namely *ROP-INTERACTIVE CRIB MOTIF-CONTAINING PROTEIN 3* (*RIC3*) and *ROP-INTERACTIVE CRIB MOTIF-CONTAINING PROTEIN 1* (*RIC1*), kinases *POLLEN RECEPTOR LIKE KINASE 6* (*PRK6*) and *LOST IN POLLEN TUBE GUIDANCE 2* (*LIP2,*) Liu et al. 2013, *CELLULOSE SYNTHASE-LIKE D1*, *AMINOPHOSPHOLIPID ATPASE 7* (*ALA7*) ATPase, *INOSITOL-POLYPHOSPHATE 5-PHOSPHATASE 12* (*5PTase12*) and DNAJ domain protein *THERMOSENSITIVE MALE STERILE 1* (*TMS1*). This supports the hypothesis, that group C transcripts are stored for later translation in pollen tubes. KEGG pathways enriched for group C transcripts were endocytosis and glycerophospholipid metabolism. For PFAM domain representation, three of 18 ANTH membrane binding domain proteins were present. Further, several genes were connected to cytoskeleton (microtubule organization), containing, for example, both developmentally regulated plasma membrane polypeptide (DREPP) family proteins MICROTUBULE-ASSOCIATED PROTEIN 18 (MAP18) and MICROTUBULE-DESTABILIZING PROTEIN 25 (MDP25), which are reported to act as actin-severing proteins in pollen tubes (Qin et al. 2014).

Group D transcripts included only 15 genes. Two of these genes play a role in the initiation of DNA replication and four are connected to regulation of DNA conformation change.

Group E transcripts comprise 85 genes and do not show enrichment for biological processes, but metabolic pathways and glutathione metabolism were enriched among KEGG pathways.

Group F transcripts comprise 589 genes. Biological process enrichment as well as KEGG pathways point to a connection to energy metabolism, represented by terms such as glutamate catabolic process, citrate metabolic process, mitochondrial ATP synthesis coupled electron transport, UDP-D-xylose biosynthetic process, glyoxylate cycle or gluconeogenesis. Further connected terms include ribosome assembly and cytoplasmic translational initiation. Identified protein domains included the ribosomal protein L12 family, ribosomal protein P1/P2, N-terminal domain and ribosomal protein L2, domain 2 and five 14-3-3 protein homologues were present.

## Discussion

We used RNA-seq to determine the transcriptomes of isolated microspores and developing pollen at four developmental stages for two *Arabidopsis* accessions. This enabled a more comprehensive qualitative and quantitative analysis, when compared with microarray analysis for the same four developmental stages (Honys and Twell 2004). Our analysis on both platforms in the Ler-0 accession was extended to include Col-0, for which we broadened our study by integrating proteomic data. This allowed us to compare pollen transcriptome dynamics between accessions and to extend the regulatory levels studied.

RNA-seq data was strongly correlated with microarray data suggesting high reproducibility of these methods. RNA-seq provided data for over 33,000 genes, a 1.5-fold increase in the number of genes compared to ATH1 arrays (Honys and Twell 2004). Among these, 6621 protein-coding genes were not previously described as male gametophyte expressed (MGE). The mean expression of these ‘new’ MGE genes was comparable to that of the whole datasets indicating unexplored roles in male gametophyte development. 606 of them were also supported by the proteome data. New MGE genes were annotated with diverse functions, rather than just a few specific groups or families, providing new information about various biological processes. Further, about 27% (1758 genes) of new MGE genes were unclassified, representing a substantial number of candidates with unknown functions.

We identified numerous long non-coding RNAs, highlighting lncRNAs as good candidates for future studies of potential regulators of pollen gene expression. Although, plant lncRNA research is limited and has focused on root development, auxin signalling or fibre development in cotton, 3 lncRNA discovered in *Oryza sativa*, *Brassica campestris* and ﻿*Zea mays* caused male sterility if under-expressed (Datta and Paul 2019). lncRNAs are also implicated in rice ovary meiosis (Li et al. 2020). In pollen, cis-acting natural antisense RNAs (*cis*-NATs) were identified in sperm and vegetative cells of *Arabidopsis* by re-analysis of published RNA-seq and microarray data (Qin et al. 2018). This study reported 1471 potential protein-coding *cis*-NAT pairs, from which 872 had at least one member expressed in pollen based on an expression threshold ≥ 1 RPM. We found an overlap of 834 genes across all pollen stages with our RNA-seq data (expression threshold > 1 TPM in at least one stage). One of the most highly expressed NATs in both datasets was At1g08727.1, which overlaps *TUA1* (At1g64740.1), a pollen-specific *ALPHA-1 TUBULIN* (Carpenter et al. 1992). NAT At1g08727.1 shows the highest expression in late pollen development stages. One lncRNA discovered in our dataset is FLORE (At1g69572), a *cis*-acting natural antisense transcript of *CYCLING DOF FACTOR 5* (*CDF5*), which forms an antagonistic pair with role in circadian regulation and flowering time (Henriques et al. 2017). FLORE may act as a late pollen regulator as it is not expressed in UNM and BCP, but it has peak expression (33 TPM) in TCP. Further examination of genes overlapping expressed natural antisense non-coding RNAs led to the discovery of proton pump interactor 1 (PPI1, At4g27500) and NAT At4g07855, both highly expressed throughout pollen development. PPI1 encodes a 14-3-3 domain protein interacting with H + ATPase. Overall, the dynamic expression of different lncRNA groups suggests functional roles in developing pollen including the modulation of protein-coding RNA expression.

### Transcriptome dynamics during pollen development

**Fig. 11:**
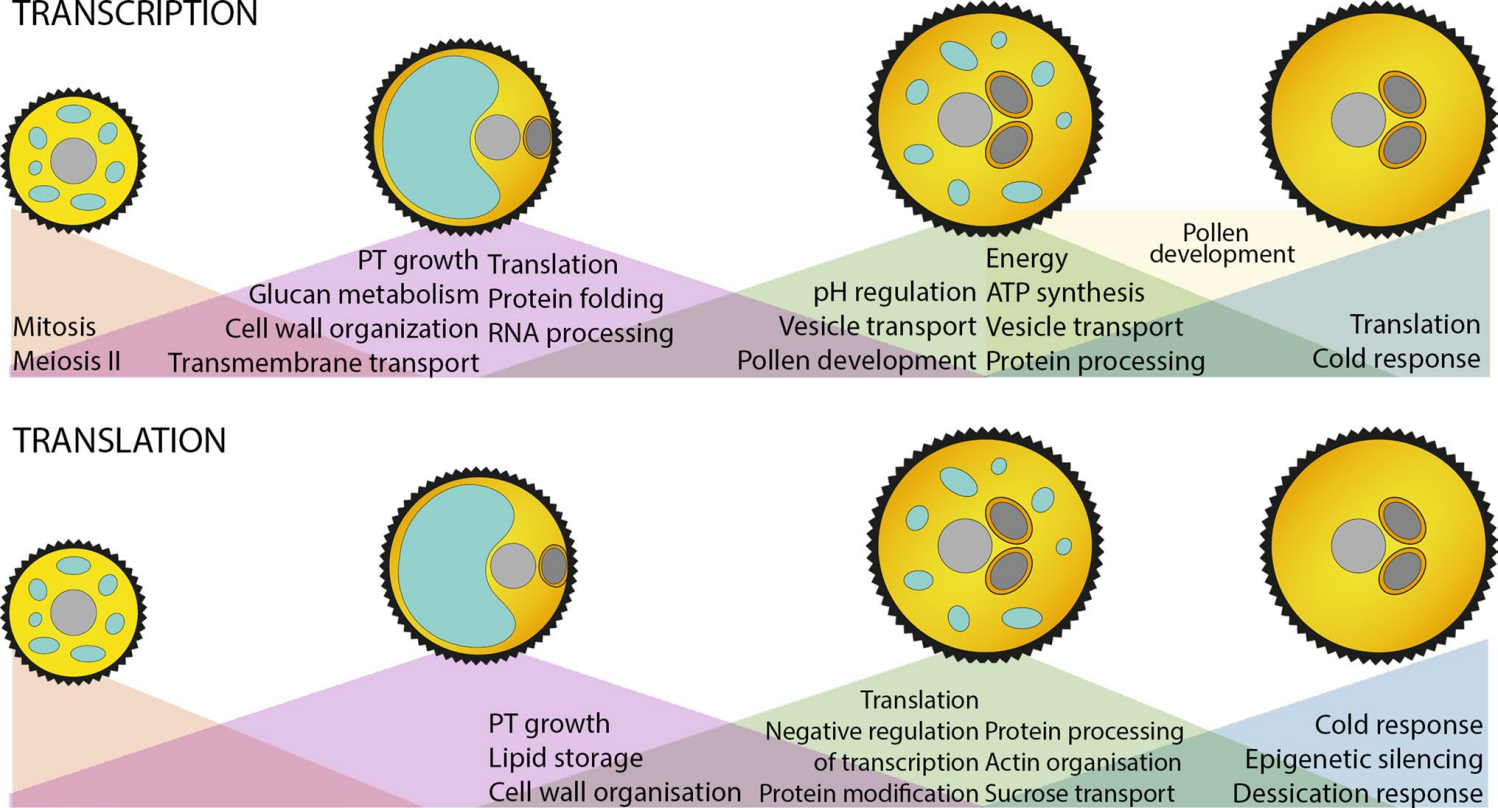
Summary of major changes in biological processes in developing *Arabidopsis* pollen. GO terms reflect the analysis of differential gene expression based on transcriptomic and proteomic data at four developmental stages. Coloured triangles indicate increased expression (left) or decreasing expression (right) of GO processes during stage transitions

RNA-seq data were analysed and compared across pollen developmental stages as well as between accessions. The differentially expressed genes were categorized by GO enrichment, KEGG pathway and String analyses. The major functional changes are summarized in Fig. 11. Briefly, UNM and BCP stages differ in the number and abundance of mitosis- and cell cycle-related transcripts. In a previous microarray analysis 61 transcripts associated with cell cycle, 45 were detected in developing pollen, with the majority expressed in UNM and BCP (Honys and Twell 2004). For Col-0 RNA-seq data, 60 of these genes were predominantly expressed in early stages, along with a further eight new cell cycle genes (Supplementary File 11). Genes that show increased expression during UNM-BCP transition include early pollen tube and cell wall organization transcripts. Transmembrane transport and glucan metabolism processes were also well represented. In accord with previous data from *Arabidopsis* (Honys and Twell 2004) and tobacco (Hafidh et al. 2018), translation initiation factors (eIFs) were mostly expressed during early stages.

Although the generative cell lineage identity is established after PMI in BCP the transcriptomes of the two early stages remain similar. This may be explained in part by the limited contribution of the generative cell to the BCP transcriptome relative to the vegetative cell (Honys and Twell 2004). Expression of several pollen tube growth and regulation-associated genes starts in the BCP, suggesting developmental priming for pollen germination. Developmental priming to prepare for either potential heat stress response or stage transition has been described during pollen development in tomato (Chaturvedi et al. 2013; Chaturvedi et al. 2016). The majority of pollen tube growth connected genes upregulated in BCP continue to increase in expression at TCP stage, therefore another explanation could be the cumulative effect of their expression throughout pollen development, as two candidates pectinesterase 5 (PME5, At2g47040) and pectinesterase 1 (PME1, At1g69940) increase nearly tenfold during UNM-BCP and BCP-TCP transitions. On the contrary, myosin 11 C2 (At1g54560) and myosin11 C1 (At1g08730), described to be essential for the high rate of pollen tube growth (Madison et al. 2015), show only a slight increase (At1g08730) or a decrease (At1g54560) in expression after BCP stage, suggesting their early expression as developmental priming.

The current study highlights the major transcriptomic switch between BCP and TCP stages consistent with previous analyses (Honys and Twell 2004; Twell et al. 2006). As a well-known example, protein synthesis genes are transcribed almost exclusively during the early phases of microgametogenesis. We also described a major decline of transcripts encoding ribosomal proteins during BCP-TCP transition which correspond to previous findings (Hafidh et al. 2018; Honys and Twell 2004). The massive synthesis of ribosomal proteins (RP) in tobacco pollen soon after the completion of PMI, is demonstrated by the abundance of RP transcripts (Bokvaj et al. 2015) and by their association with polysomes (Hafidh et al. 2018). This highlights active translation at this stage and persistence of the translation apparatus during late pollen development and the progamic phase (Hafidh et al. 2018; Hafidh and Honys 2021). The profiling and distribution of RP transcripts and proteins identified in this study suggest that the same mechanism of RP translation during early pollen development is active in *Arabidopsis*.

The start of the late developmental phase (TCP) is marked by up-regulation of transcripts involved in vesicular transport, pH regulation and energy metabolism. These transcripts may have roles later in pollen tube growth and regulation, where vesicular transport and formation of a pH gradient are essential for rapid PT growth. In addition, the number of transcripts annotated as ‘pollen developmental’ increases steadily from early to later stages. Some transcripts encoding pollen tube regulatory proteins are predominantly expressed in MPG stage and appear to represent a specific class of late pollen tube mRNAs.

The final developmental stage is distinguished by an increase in the expression of stress response genes including cold and salt stress related proteins, which probably play protective role during the desiccation process of angiosperm pollen which typically undergoes dehydration and developmental arrest prior to dispersal (Franchi et al. 2011). In seeds, the major stress-like condition accompanying dehydration can cause DNA damage including strand breakage or telomeric shortening (Osborne and Boubriak 2002). Among DEGs detected during TCP-MPG transition there were 53 genes involved in DNA repair, including DNA ligase 1 responsible for double-strand breakage repair and the telomerase maintenance genes HOMOLOG OF X-RAY REPAIR CROSS COMPLEMENTING 3 (XRCC3) and RAD50 (Gallego and White 2001; Bleuyard and White 2004).

Protein products of genes upregulated in MPG were distinctively associated with plasma membrane, cell wall and apoplast. These compartments play important roles in pollen germination and in pollen tube guidance, reception, and fertilization, through signalling and crosstalk with female reproductive tissues (Hafidh et al. 2016; Hafidh and Honys 2021; Johnson et al. 2019), cell wall remodelling or stress response (reviewed in Ge et al. 2011). The most prevalent stress-related group, accounting for 60 genes upregulated in MPG, are genes responsive to drought tolerance. Three DELLA repressor genes are upregulated during TCP-MPG transition. In general DELLA proteins work as growth repressors, while DELLA-3 is reported to play role in protein storage in seeds (Hu et al. 2021). Although pollen dehydration is a crucial phase in maturation the underlying mechanisms are not well described, and this study could provide suitable candidate genes for further studies.

Apart from the general trends, we focused on a few selected groups. We categorized transcription factor families and the data are supported by previous findings. For example, 17 MADs-box genes were reported to be expressed in mature pollen (Pina et al. 2005), 16 of which were present in our MPG datasets. Similarly, our results follow the observed divergent expression of TFs, describing early, constitutive, and late TFs (Honys and Twell 2004). The V5 plant transcription factors database (PTFD) (Mitsuda and Ohme-Takagi 2009), which was used for annotation of the RNA-seq data, accounts for 127 bZIP and 225 bHLH TFs, an increase of 1.7-fold in each case. Thus, our data substantially extends knowledge of TF expression. Similarly, ribosomal, stress-related, cell wall-connected and other transcript group were sorted according to temporal expression enabling examination of any of these groups of genes.

When we examined transcriptome differences between Col-0 and Ler-0, although around 3,000 DEGs were found at each stage, no major transcript groups or pathways differed between accessions. The observed differences in expression of individual genes may therefore be genotype-dependent and influenced by the experimental conditions. Although there is limited information about differences in pollen gene expression between *Arabidopsis* accessions, we observed reduced levels of the *AtSUC1* transcripts in Ler-0 compared with Col-0, consistent with reduced immunodetection of AtSUC1 in mature pollen of Ler and C24 ecotypes (Feuerstein et al. 2010). In future, studying protein abundance of sucrose transporters between *Arabidopsis* accessions could prove interesting for deciphering ecotype differences in pollen tube germination.

### Identification of transcript isoform switches during pollen development

The differential usage of transcript isoforms is common in plants, with around 22% of AS events resulting in changes to protein sequences in *Arabidopsis* (Vaneechoutte et al. 2017). Among AS events, intron retention is the most frequent, but AS can also result in protein sub-functionalization (Ner-Gaon et al. 2004). Keller et al. 2017 reported 5000 to 8000 genes showing intron retention during pollen development for two tomato cultivars. Intron retention was also reported to be the predominant AS mechanism in developing pollen of *Brassica rapa* (Golicz et al. 2021). Further, intron retention was found to be stage-specific, similar to our findings in *Arabidopsis*, and IR may thus represent an important mechanism of functional attenuation of related subsets of genes (Golicz et al. 2021). In a previous analysis, isoform usage between mature pollen and seedlings of *Arabidopsis* was reported to be similar, with few pollen-specific changes that were mostly connected to transcripts involved in splicing (Loraine et al. 2013). In our more comprehensive study, numerous transcript isoforms were detected that varied between UNM-BCP and BCP-TCP transitions. Their categorization highlighted processes that were diversified by mRNA processing, including splicing. In all stage comparisons, differential isoform usage was discovered in a variety of genes, including transporters, transcription factors, enzymes, heat stress proteins or ribosomal proteins. The functional consequences of isoform switching included loss or gain of signal peptide, domain presence or change in NMD sensitivity. In UNM-BCP, the switches may be connected to the regulation of PMI. Relevant candidates include kinesin-like proteins KIN12B (At3g23670) and KIN-12A (At4g14150) which link microtubules in the phragmoplast during plant cell division (Vanstraelen et al. 2006). It suggests IR to be an effective way to switch off cytokinetic transcripts after PMI1. The highest frequency of isoform switching was detected during BCP-TCP transition when differential expression changes are also maximum. The genes with DIU in this stage transition were enriched for processed connected to mRNA and protein processing. Also, vesicle transport and GTPase activity connected genes were enriched suggesting a regulation of transcripts of proteins required for the rapid pollen tube growth. In the TCP to MPG transition, we observed enrichment for transporters. In summary, pollen development is accompanied by numerous changes in isoform usage specific for each developmental phase.

Although differences in AS events were less frequent between Col-0 and Ler-0, hundreds of switches were present at each stage. Despite originating from the same parental lineage, these accessions show differences in physiological and morphological traits (Passardi et al. 2007). The reported changes in isoform usage could provide leads for understanding the differences in gene regulation between these accessions. For example, a different dominant isoform of UBQ14 and UBQ4 are present in each accession at TCP and MPG stages, and both ubiquitins influence development and environmental response (Sun and Callis 1997). Another candidate with DIU is VOLTAGE DEPENDENT ANION CHANNEL 5 (VDAC5), which lacks a porin domain I in Ler-0 and the dominant isoform is NMD sensitive (Fig. 8E). *VDAC5* is reported to carry single nucleotide polymorphisms in geographically restricted populations of the Iberian Peninsula, indicating potential involvement in environmental adaptation (Tabas-Madrid et al. 2018). However, analysis of AS in other plant organs will be needed to provide a perspective of its potential contribution to the phenotypic differences between these accessions.

### Proteomic dynamics during pollen maturation

Previous proteomic studies have described the major portion of the *Arabidopsis* mature pollen proteome to be involved in protein synthesis, cytoskeleton organization, metabolism, cellular transport and signalling (Ge et al. 2011; Grobei et al. 2009). Transcripts involved in energy metabolism and protein synthesis were reported to be either directly translated at high rate (energy) or transcribed in early stages and translated during late stages (Holmes-Davis et al. 2005). Our developmental proteomic data support and extend these findings by integration with transcriptomic data. Proteome changes during UNM-BCP are the least dramatic and involve cell wall organization, lipid storage and pollen tube developmental (Fig. 11). In accord with transcriptome results, the main shifts in protein synthesis occur during BCP-TCP transition, with TCP as the start point for translation of the protein synthesis machinery and the reduction of transcript abundance. Cytoskeleton organization proteins are also enriched in the later developmental stages.

In the MPG proteome, stress tolerance and epigenetic modification processes are over-represented compared to previous stages. Male gametophyte development involves extensive epigenetic reprogramming (Ashapkin et al. 2019; Borg et al. 2020) and our data show high levels of accumulation of AGO1 (At1g48410) and NRPD1B (At2g40030) in mature pollen. These proteins are active in 21nt siRNA production in the vegetative nucleus. Interestingly, despite the limited contribution of the sperm cell proteome, we also detected increased expression of AGO5 (At2g27880) and AGO9 (At5g21150), a component of the Argonaute complex responsible for de novo methylation via RNA directed DNA methylation and probably siRNA-mediated transposon silencing in sperm cells (Ashapkin et al. 2019). The early peak in transcripts for *AGO1*, *NRPD1B* and *AGO9* (*AGO5* RNA peaks in TCP), highlight these as examples of transcripts with delayed translation during pollen development.

We compared our developmental pollen proteome data with major studies in *Solanum lycopersicum* (Chaturvedi et al. 2013) and *Nicotiana tabacum* (Ischebeck et al. 2014). In tomato 1104 proteins were detected in mature pollen and for proteins well conserved between tomato and *Arabidopsis* (> 90% NCBI BLAST similarity scores), we identified 194 homologous *Arabidopsis* genes. Among these, there was an overlap of 70.6% (137 proteins) with our developmental proteome and 130 had riBAQ values higher than 1 in mature pollen, with 255 on average. The tobacco study described 3888 proteins from 8 developmental stages spanning male meiocytes to pollen tubes (Ischebeck et al. 2014). For the 1478 unique tobacco proteins with a homologue in the *Arabidopsis*, 1348 proteins were identified in our analysis, highlighting the similarity between proteomes of developing pollen of these two species. In total, 111 proteins were shared between *Arabidopsis*, tobacco, and tomato (Supplementary File 12). In summary, *Arabidopsis* pollen developmental proteomes show high similarity with published proteome data for mature pollen of *Arabidopsis* (Grobei et al. 2009)*,* tobacco and tomato. In addition, our proteomic analysis identified 2150 proteins, which have not been previously detected in the male gametophyte (Supplementary File 13).

### Coupling of transcriptome and proteome data reveals mRNA fate in pollen development

Post-transcriptional regulation of mRNAs in developing pollen is a crucial process for understanding male reproductive development. Apart from mRNA storage, there have been several attempts to decipher the relationship between transcription and translation in developing pollen. In previous work (Honys and Twell 2004) and in this article, we showed that most transcripts responsible for PT organization appear at bicellular stage. These transcripts are stored to allow rapid translation during pollen activation and germination (Hafidh et al. 2018). Previous comparisons of *Arabidopsis* MPG proteomic data with transcriptomic data have uncovered inverse relationships between mRNA and protein abundance (Holmes-Davis et al. 2005). These included energy-related genes in contrast to cell wall organization genes where mRNAs were more abundant relative to proteins (Holmes-Davis et al. 2005). They suggested that the energy-related proteins are stored in advance of resumption of metabolic activities upon pollen activation, whereas cell wall proteins are translated to support PT growth. Similarly, transcripts with direct and delayed modes of translation in developing pollen of tomato were suggested based on transcriptomic and proteomic data (Keller et al. 2018).

We addressed mRNA fate and the timing of translation separately by clustering developmental proteome and transcriptome data. Using k-means clustering, we divided the genes among seven groups defined by the relationship between gene expression and translation. The most abundant group consisted of stably transcribed and translated energy and metabolism connected genes. Early-stage synthesized proteins were mainly focused on replication. Genes with delayed translation or with stored transcripts are connected to RNA processing and cytoskeleton organization. The later may account for the pool of stored mRNA to support rapid PT growth upon pollen germination (Hafidh et al. 2016; Honys and Twell 2004; Rutley and Twell 2015). The direct link between transcription and translation during pollen development could be further addressed by studying the translatome of active polysomes and monosomes. This approach has been reported for in vitro and in vivo pollen tubes (Lin et al. 2014) including a recent study of the heat stress response (Poidevin et al. 2020).

## Conclusions

We analysed transcriptome and proteome dynamics accompanying four developmental stages of microgametogenesis in the Col-0 and Ler-0 accessions of *Arabidopsis*. RNA-seq and up to date genome annotation enabled us to extend the coverage and resolution of previous microarray analyses qualitatively and quantitatively. We demonstrated high reproducibility and comparability of both transcriptomic platforms. RNA-seq also allowed us to detect thousands of lncRNAs and their dynamics as potential regulators of pollen development. Similarly, we described numerous alternative splicing events in developing pollen and identified candidate transcripts regulated predominantly by intron retention. To understand mRNA fate and translation dynamics, we compared transcriptomic and proteomic data and proposed transcript groups based on their potential temporal translation. Overall, this work provides an integrated perspective of gene expression dynamics in developing pollen and a foundation for exploration of the role of alternative splicing in the male gametophyte of *Arabidopsis thaliana*.

## Methods

### Plant cultivation and isolation of microspores and developing pollen

Plants of *Arabidopsis thaliana* accession Columbia-0 (Col-0) and Landsberg erecta (Ler-0) were grown in controlled-environment chambers at 22 °C with a 16-h photoperiod and illumination of 150 μmol/m^2^/sec. Mature pollen grains (MPG) were collected either with a modified vacuum cleaner using 100, 53 and 5 μm mesh and/or by shaking of cut inflorescences of 5- to 6-week-old plants in 0.1 M mannitol as described (Duplakova et al. 2016). Populations of uninucleate microspores (UNM), bicellular pollen (BCP) and tricellular pollen (TCP) were released from anthers of closed flower buds and separated by Percoll density gradient centrifugation as described (Duplakova et al. 2016; Honys and Twell 2004).

### RNA extraction, cDNA library preparation and sequencing

Total RNA was isolated from populations of isolated microspores or developing pollen (UNM, BCP, TCP and MPG) using the RNeasy Plant Kit (Qiagen) following the manufacturer’s instructions. RNA was Dnase-treated (DNA-free™ Kit Ambion, Life Technologies) according to the manufacturer’s protocol. The yield and purity of RNA were determined spectrophotometrically using an Agilent 2100 Bioanalyzer. A slightly modified SmartSeq2 protocol was used to synthesize cDNA from poly(A) + RNA with an oligo(dT)-tailed primer (Picelli et al. 2013, 2014). A low-input Nextera protocol (Baym et al. 2015) was used to prepare the final libraries, which were sequenced on a NextSeq500 instrument with single-end 75 bp read length (SE75).

### RNA-seq data processing, mapping and assembly of reads

The quality of single-end raw reads was revised by FastQC ver. 0.11.8 (Wingett and Andrews 2018), and Cutadapt ver. 1.9.1 (Martin 2011). The quality reads (phred score > 20) were trimmed of technical sequences using the same Cutadapt software and mapped to the *A. thaliana* reference genome (ver. TAIR10) downloaded from Araport (Pasha et al. 2020) with STAR ver. 206.1a (Dobin et al. 2013). The gtf annotation file obtained from Araport was used for STAR index preparation. Gene and isoform counts (including normalized TPM values) were acquired with RSEM (Li and Dewey 2011). The expression threshold for RNA-seq data was set to 3 TPM (Transcripts Per Kilobase Million) for each biological replicate. The data were imported into Rstudio with Tximport (Soneson et al. 2015) and processed further. For differential expression analysis DESeq2 ver. 3.8 (Love et al. 2014) was used with adjusted *p* values < 0.05 and FoldChange ≥  ± 2 as thresholds for establishing differentially expressed genes (DEGs). For analysis of differential exon usage, DexSeq (Anders et al. 2012; Reyes et al. 2013) was used with the STAR transcriptome alignment as input. To obtain differential isoform usage the isoform level quantification output of RSEM was processed by IsoformSwitchAnalyser v. 4.1 (Vitting-Seerup and Sandelin 2017; Vitting-Seerup et al. 2019). The thresholds for both gene and isoform expression ware set to 3 (function PreFilter). Adjusted *p* values of < 0.05 and log_2_ fold change > 1 (diF > 0.1 for DIU) were used as statistically significant thresholds in both analyses. Domain switch consequences were analysed according to Pfam v. 33.1 (Mistry et al. 2021) and coding potential was established with CPC2 calculator (Kang et al. 2017). Signal peptide presence was analysed with SignalP-5.0 (Almagro Armenteros et al. 2019).

### Comparison of ATH1 data and RNA-seq data

Affymetrix ATH1 genome array data were MAS5 normalized with the exclusive approach, such that expressed genes have a present detection call in both replicates (Duplakova et al. 2016; Honys and Twell 2004). MAS5 normalized hybridization signals from expressed genes were compared to normalized TPM values for RNA-seq data. Any ambiguous probe sets representing gene models unique to ATH1 arrays were removed from our analyses (Supplementary File 14).

### Protein extraction and LC–MS/MS analysis

Proteins were isolated from UNM, BCP, TCP and MPG using TRI Reagent solution (Sigma-Aldrich, product No. T9424) following the manufacturer’s instructions. Individual protein samples were processed by filter-aided sample preparation (FASP) with modifications. The samples were mixed with 8 M UA buffer (8 M urea in 100 mM Tris–HCl, pH 8.5), loaded onto the Microcon device with MWCO 30 kDa (Merck Millipore) and centrifuged at 7000 × *g* for 30 min at 20 °C. The retained proteins were washed (all centrifugation steps after sample loading done at 14,000 × *g*) with 200 μL UA buffer. The final protein concentrates kept in the Microcon device were mixed with 100 μL of UA buffer containing 50 mM iodoacetamide and incubated in the dark for 20 min. After the next centrifugation step, the samples were washed three times with 100 μL UA buffer and three times with 100 μL of 50 mM NaHCO_3_. Trypsin (sequencing grade, Promega) was added onto the filter and the mixture was incubated for 18 h at 37 °C (enzyme:protein ratio 1:100). The tryptic peptides were finally eluted by centrifugation followed by two additional elutions with 50 μL of 50 mM NaHCO_3_. Directly after FASP, peptides were extracted into LC–MS vials by 2.5% formic acid (FA) in 50% acetonitrile (can) and 100% ACN with addition of polyethylene glycol (20,000; final concentration 0.001%) and concentrated in a SpeedVac concentrator (Thermo Fisher Scientific) prior LC–MS analyses. LC–MS/MS analyses of all peptide mixtures were done using Ultimate 3000 RSLCnano system (SRD-3400, NCS-3500RS CAP, WPS-3000 TPL RS) connected to Orbitrap Elite hybrid mass spectrometer (Thermo Fisher Scientific). Prior to LC separation, tryptic digests were online concentrated and desalted using trapping column (100 μm × 30 mm) filled with 3.5-μm X-Bridge BEH 130 C18 sorbent (Waters). After washing of the trapping column with 0.1% FA, peptides were eluted (flow rate -300 nl/min) onto an analytical column (Acclaim Pepmap100 C18, 3 µm particles, 75 μm × 500 mm; Thermo Fisher Scientific) with a 100 min nonlinear gradient program (1–56% of mobile phase B; mobile phase A: 0.1% FA in water; mobile phase B: 0.1% FA in 80% ACN). Equilibration of the trapping column and the column was done prior to sample injection to sample loop. The analytical column outlet was directly connected to the Digital PicoView 550 (New Objective) ion source with sheath gas option and SilicaTip emitter (New Objective; FS360-20-15-N-20-C12) utilization. ABIRD (Active Background Ion Reduction Device, ESI Source Solutions) was installed.

MS data were acquired in a data-dependent strategy selecting up to top 10 precursors based on precursor abundance in the survey scan (350–2000 m*/z*). The resolution of the survey scan was 60 000 (400 m*/z*) with a target value of 1 × 10^6^ ions, one microscan and maximum injection time of 200 ms. HCD MS/MS (32% relative fragmentation energy) spectra were acquired with a target value of 50 000 and resolution of 15 000 (at 400 m*/z*). The maximum injection time for MS/MS was 500 ms. Dynamic exclusion was enabled for 45 s after one MS/MS spectra acquisition and early expiration was disabled. The isolation window for MS/MS fragmentation was set to 2 m*/z*.

### Processing of proteomic data

The analysis of the mass spectrometric RAW data files was carried out using MaxQuant software (version 1.6.0.16). MS/MS ion searches were conducted against the modified cRAP database (The common Repository of Adventitious Proteins, cRAP database) containing protein contaminants such as keratin and trypsin, and the UniProtKB protein database for *Arabidopsis thaliana* (see ref. UniprotKB for the ftp server address, the number of protein sequences was 27,567). Default precursor and fragment mass tolerances were used with software MS data recalibration enabled. Oxidation of methionine and proline, deamidation (N, Q) and acetylation (protein N-terminus) as optional modification, carbamidomethylation (C) as fixed modification and one enzyme miss cleavage were set. Peptides and proteins with FDR threshold < 0.01 and proteins having at least one unique or razor peptide were reported by MaxQuant only. Match between runs among all analyses and second peptide identification features of MaxQuant were enabled. Protein abundance was assessed using protein intensities calculated by MaxQuant. Limma R package was used for protein group intensities normalization (loessF) and statistical significance testing of differences between individual stages. Differently expressed proteins (DEPs) were ascertained based on the limma results as follows: (1) protein groups having log2 fold change >|1|, adjusted p value (Benjamini–Hochberg procedure) < 0.05 and quantified in at least 2 replicates were considered as quantitatively changing between the stages compared; (2) qualitative changes were considered separately and contained protein groups being quantified in at least two replicates of one stage and absent in the other one.

### Annotation and enrichment analyses

The lists of DEGs and DEPs were annotated with gene names and symbols derived from ThaleMine v5.0.2 (Pasha et al. 2020). GO Enrichment for biological processes, cellular content and molecular functions was analysed by Panther16.0 (Thomas et al. 2003) with Fisher’s Exact test with False discovery rate (FDR) correction. ReviGo (Supek et al. 2011) was used to summarize GO enrichment analyses and to visualize the top enriched terms. The results were further visualized in MapMan v. 3.5.1R2 (Thimm et al. 2004) Enriched KEGG pathways were calculated and rendered with PathView (Luo et al. 2009, 2017) using default settings. Data processing and sorting was executed in Microsoft Excel and RStudio with ggplot2, venn.diagram and enhanced volcano R packages used for plots rendering.

### k-means clustering

log2 scaled RiBaq values for proteome analysis and log2 scale TPM values for transcriptome analysis were used as input data. Only genes present in both analyses were processed. The number of clusters suitable for the size of the datasets was determined with the Elbow method. This resulted in the division of both datasets into nine clusters according to expression pattern across the four developmental stages. K-means clustering was calculated in R with the tidyverse and maggrire packages.

## Supplementary Information

Below is the link to the electronic supplementary material.

Supplementary file1 (XLSX 5070 kb)

Supplementary file2 (XLSX 6685 kb)

Supplementary file3 (XLSX 107 kb)

Supplementary file4 (XLSX 50 kb)

Supplementary file5 (XLSX 1015 kb)

Supplementary file6 (XLSX 559 kb)

Supplementary file7 (XLSX 153 kb)

Supplementary file8 (XLSX 66 kb)

Supplementary file9 (XLSX 586 kb)

Supplementary file10 (XLSX 40 kb)

Supplementary file11 (XLSX 5336 kb)

Supplementary file12 (XLSX 17 kb)

Supplementary file13 (XLSX 208 kb)

Supplementary file14 (XLSX 29 kb)

Supplementary file15 (PDF 20 kb)

## Data Availability

The RNA-seq data generated and analysed during the current study are available in ArrayExpress with accession code E-MTAB-9456. The proteome data generated and analysed are available in PRIDE with accession code PXD033305. Affymetrix data analysed during this study are included in Honys and Twell (2004) (https://doi.org/10.1186/gb-2004-5-11-r85) Web-based queries of the RNA-seq data can be made at the EVOREPRO database (www.evorepro.plant.tools) as described in Julca et al. 2021. (https://doi.org/10.1038/s41477-021-00958-2).
