# Correction: Regulatory dynamics of gene expression in the developing male gametophyte of *Arabidopsis*

**DOI:** 10.1007/s00497-023-00471-w

**Published:** 2023-06-15

**Authors:** Božena Klodová, David Potěšil, Lenka Steinbachová, Christos Michailidis, Ann-Cathrin Lindner, Dieter Hackenberg, Jörg D. Becker, Zbyněk Zdráhal, David Twell, David Honys

**Affiliations:** 1 Institute of Experimental Botany of the Czech Academy of Sciences, Rozvojová 263, 165 02 Prague 6, Czech Republic; 2 Department of Experimental Plant Biology, Faculty of Science, Charles University, Viničná 5, Praha 2, 128 00 Czech Republic; 3 Mendel Centre for Plant Genomics and Proteomics, Central European Institute of Technology, Masaryk University, Kamenice 5, 625 00 Brno, Czech Republic; 4 Instituto de Tecnologia Química e Biológica António Xavier, Universidade Nova de Lisboa (ITQB NOVA), Av. da República, 2780-157 Oeiras, Portugal; 5 Department of Genetics and Genome Biology, University of Leicester, Leicester, LE1 7RH UK; 6 Instituto Gulbenkian de Ciência, Rua da Quinta Grande 6, 2780-156 Oeiras, Portugal; 7 National Centre for Biomolecular Research, Faculty of Science, Masaryk University, Kamenice 5, 625 00 Brno, Czech Republic; 8 KWS SAAT SE & Co. KGaA, Grimsehlstraße 31, 37574 Einbeck, Germany

**Correction to: Plant Reproduction** 10.1007/s00497-022-00452-5

The article Regulatory dynamics of gene expression in the developing male gametophyte of Arabidopsis written by Božena Klodová, David Potěšil, Lenka Steinbachová, Christos Michailidis, Ann‑Cathrin Lindner, Dieter Hackenberg, Jörg D. Becker, Zbyněk Zdráhal, David Twell and David Honys originally published Online First without Open Access.

After online publication the author decided to opt for Open Choice and to make the article an Open Access publication. Therefore, the copyright of the article has been changed to ©The Author(s) and the article is forthwith distributed under the terms of the Creative Commons Attribution.

