# Supplementary figures and images for: Regulatory dynamics of gene expression in the developing male gametophyte of Arabidopsis
Source: Plant Reprod. 2022 Oct 25;36(3):213–41. doi: 10.1007/s00497-022-00452-5 (PMC10363097; doi:10.1007/s00497-022-00452-5)

GO biological processes

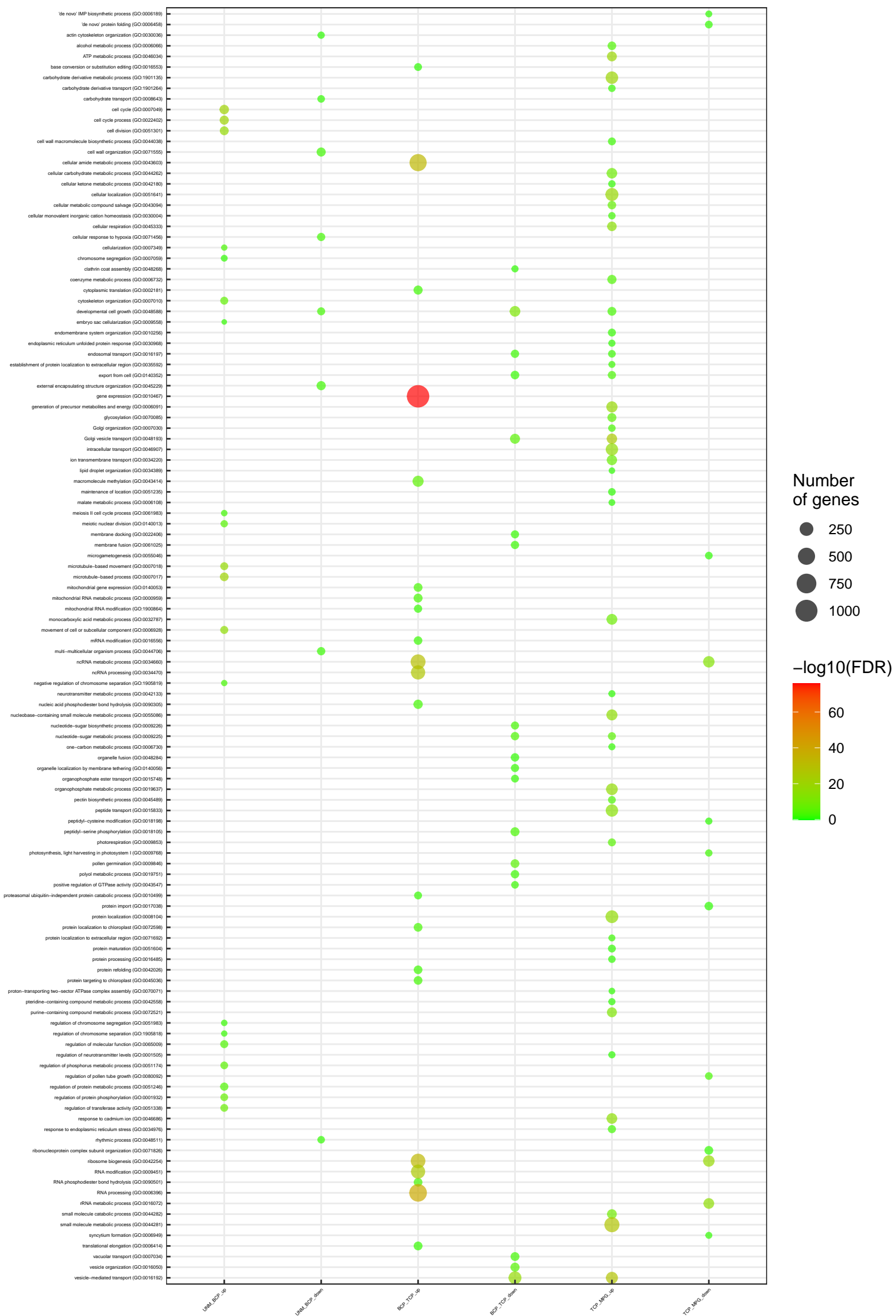

Supplement: Supplementary file 15 — Supplementary file15 (PDF 20 kb) [file 497_2022_452_MOESM15_ESM.pdf]
